# UBQLN2 links proteotoxicity with lipid metabolism in neurodegeneration

**DOI:** 10.1038/s41593-026-02226-y

**Published:** 2026-03-30

**Authors:** Yang Liu, Zhiyuan Huang, Yu-Wen Hsu, Pragney Deme, Ashley M. Frankenfield, Suheng Wu, Xiaofeng Zhao, Honghe Liu, Tao Zhang, Elizabeth J. Alexander, Mingming Liu, Yanjun Zhang, Haocheng Wang, Yixin Zhou, Mervyn J. Monteiro, Ling Hao, Norman J. Haughey, Jiou Wang

**Affiliations:** 1 Department of Biochemistry and Molecular Biology, Bloomberg School of Public Health, Johns Hopkins University, Baltimore, MD USA; 2 Department of Neuroscience, School of Medicine, Johns Hopkins University, Baltimore, MD USA; 3 Department of Physiology, School of Medicine, Tulane University, New Orleans, LA USA; 4 Department of Chemistry, George Washington University, Washington, DC USA; 5 Department of Neurobiology, University of Maryland, Baltimore, MD USA; 6 Department of Chemistry & Biochemistry, University of Maryland, College Park, MD USA

**Keywords:** Lipids, Amyotrophic lateral sclerosis, Mechanisms of disease

## Abstract

Protein homeostasis and lipid metabolism are essential processes frequently disrupted in neurodegenerative diseases. However, their mechanistic intersection in disorders such as amyotrophic lateral sclerosis (ALS) and frontotemporal dementia (FTD) remains unclear. Ubiquilin 2 (UBQLN2) is a protein quality control factor linked to ALS/FTD. Through multi-omic analyses of induced pluripotent stem cell (iPSC)-derived neurons harboring disease-associated UBQLN2 mutations, we uncovered UBQLN2 as a molecular hub linking lipid dysregulation and proteostasis, the perturbation of which contributes to neurodegeneration. UBQLN2 mediated the degradation of ILVBL (acetolactate synthase-like protein) and ALDH3A2 (aldehyde dehydrogenase 3 family member A2), two enzymes essential for mitochondrial lipid catabolism associated with lipid droplets and neuronal viability. ALS/FTD-linked UBQLN2 mutations and TAR DNA-binding protein 43 (TDP-43) pathology impair the degradation of ILVBL and ALDH3A2, leading to metabolic dysfunction and neurodegeneration. Restoring the UBQLN2–ILVBL/ALDH3A2 axis attenuates neurodegenerative phenotypes in neurons, organoids and mice, establishing UBQLN2 as a critical regulator of metabolic homeostasis in ALS/FTD and other related neurodegenerative diseases.

## Abstract (web summary)

ALS/FTD-linked mutations in UBQLN2, a protein quality control factor, impair degradation of enzymes essential for mitochondrial lipid catabolism, leading to metabolic dysfunction and neurodegeneration.

## Main

Neurodegenerative diseases such as ALS and FTD feature not only impaired proteostasis but also dysregulated lipid metabolism^1,2^. ALS involves progressive motor neuron loss, and FTD is the second most common cause of early-onset dementia^3^. Despite distinct clinical manifestations, ALS and FTD share overlapping genetic etiologies and pathological hallmarks and are referred to as a disease spectrum^3^. A common feature is the presence of ubiquitin-positive and TDP-43-positive proteinaceous inclusions^4,5^. Cytoplasmic mislocalization and aggregation of TDP-43 are observed in most ALS cases (approximately 97%), in nearly half of FTD cases (approximately 45%) and in a subset of Alzheimer’s disease cases^6–8^.

In addition to the proteinopathies, metabolic disturbances are pervasive in ALS/FTD. In patients with ALS/FTD and models, neuronal and glial glucose metabolism is perturbed^1,2,9–15^, and mitochondrial dysfunction contributes to chronic energy stress^1,16^. Under glucose deprivation, cells reprogram energy metabolism toward lipids, accompanied by autophagic membrane digestion and lipid droplet (LD) biogenesis as a compensatory energy source^17–20^. Emerging evidence suggests aberrant lipid metabolism in the pathogenesis of ALS/FTD linked to mutations in genes such as C9orf72, SOD1, TDP-43 and GRN^21–24^. For example, GRN mutations perturb lysosomal lipid processing, affecting phospholipid turnover and cholesterol ester regulation^24^. Nevertheless, the molecular mechanisms linking proteinopathies, impaired glucose metabolism and lipid metabolic reprogramming in ALS/FTD remain incompletely understood.

UBQLN2, a ubiquitin-binding shuttle protein, is genetically linked to familial and sporadic ALS/FTD^25,26^. UBQLN2 contains an amino-terminal ubiquitin-like (UBL) domain and a carboxyl-terminal ubiquitin-associated (UBA) domain that facilitate the delivery of polyubiquitinated proteins to the proteasome^27^. ALS/FTD-associated mutations mainly cluster within a proline-rich PXX repeat region unique to UBQLN2 among the ubiquilin family^25,28,29^. UBQLN2 has been observed in various neurodegenerative proteinaceous inclusions, including misfolded TDP-43 and C9orf72-derived dipeptide repeat proteins^25,30–33^. UBQLN2-positive aggregates are also observed in non-ALS/FTD proteinopathies, including synucleinopathies and polyglutamine disorders^34,35^. Beyond its canonical role in proteostasis, recent studies implicate UBQLN2 in diverse cellular functions, including stress granule dynamics, mitochondrial quality control and axonal remodeling^30,36–38^.

Here we identify UBQLN2 as a critical link between proteostasis and lipid metabolism, acting through two key client enzymes, ILVBL and ALDH3A2. This UBQLN2–ILVBL/ALDH3A2 axis is disrupted by disease-associated UBQLN2 mutations or TDP-43 proteotoxicity, resulting in lipid metabolic reprogramming that contributes to the pathogenesis of ALS/FTD and related neurodegenerative diseases.

## Results

### UBQLN2 mutations prolong protein half-lives and disrupt metabolic homeostasis

**Fig. 1:**
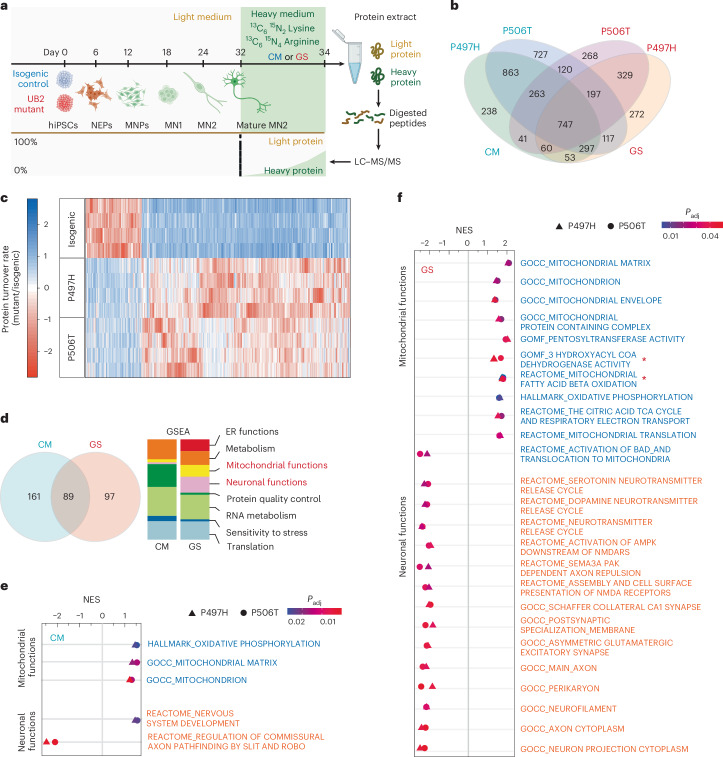
Global proteomic turnover profiling reveals metabolic remodeling in UBQLN2 mutant motor neurons under energy stress. **a**, Schematic overview of the experimental workflow for measuring proteomic half-lives in isogenic and UBQLN2 mutant iPSC-derived iMNs cultured in either CM, representing the basal condition, or glucose-free medium, representing the GS condition. Neurons were pulsed with heavy isotope-labeled amino acids, and light/heavy protein ratios were quantified by LC–MS/MS to assess turnover rates. **b**, Venn diagrams show the significantly altered protein half-lives (*P*_adj_ < 0.05) in P497H and P506T mutant iMNs under CM or GS. **c**, Heatmap of 1,333 proteins with significantly altered turnover (out of 6,326 quantified), with the majority (1,058 proteins) exhibiting prolonged half-lives in mutant iMNs. Values were converted to *z*-scores. **d**, Significantly enriched signal pathways (*P*_adj_ < 0.05) in mutant iMNs under CM and GS revealed by GSEA of proteomic half-lives, highlighting the biological processes affected in UBQLN2 mutant neurons under both conditions. Multiple hypothesis-testing corrections were applied using the Benjamini–Hochberg FDR procedure (two-sided). **e**,**f**, GSEA identified consistent enrichment of mitochondrial functional pathways (blue) and loss of neuronal functions (red) in mutant neurons under CM (**e**) and GS (**f**) conditions. Statistical analysis for proteomics was conducted as follows: one-way ANOVA was performed to assess differences in half-lives among the three groups (isogenic control, UBQLN2-P497H and UBQLN2-P506T, *n* = 4 biological replicates from two independent experiments). To correct for multiple comparisons across the proteome, *P* values were adjusted using the Benjamini–Hochberg procedure. ER, endoplasmic reticulum. Schematic in **a** created in BioRender; Liu, Y. https://biorender.com/2fe5mp9 (2025).

**Extended Data Fig. 1:**
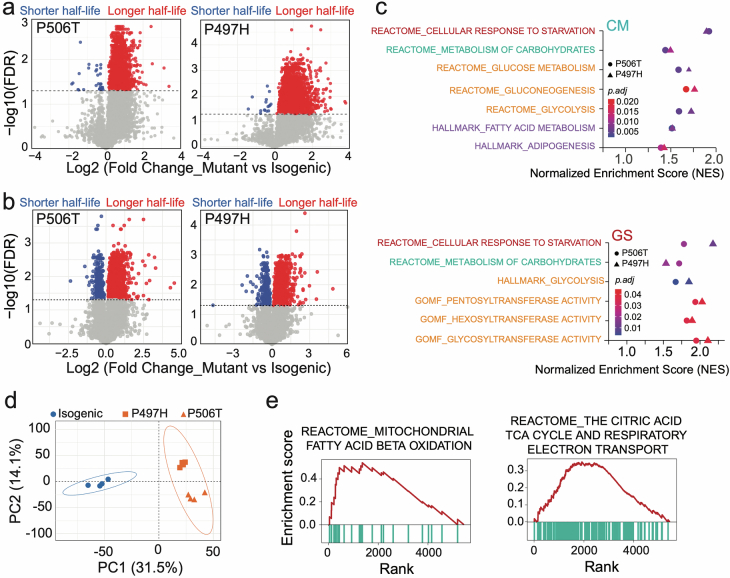
Proteostasis perturbation in UBQLN2 mutant neurons reflects metabolic remodeling. **a** and **b**, Volcano plots show differentially regulated protein half-lives in P506T and P497H UBQLN2 mutant iMNs compared to isogenic controls, under the condition of either complete medium (CM, **a**) or glucose starvation (GS, **b**) for two days. A substantial fraction of the proteome displays significantly altered turnover (adj_p < 0.05), with more proteins exhibiting prolonged half-lives (red) than shortened ones (blue). **c**, Gene set enrichment analysis (GSEA) identified proteostasis- and metabolism-related pathways significantly enriched among proteins with altered turnover in UBQLN2 mutant iMNs under CM or GS conditions (adj_p < 0.05). NES = normalized enrichment score; the dot size and color represent statistical significance and enrichment direction, respectively. **d**, Principal component analysis (PCA) of proteomic turnover under GS distinguishes isogenic controls from UBQLN2 mutant neurons. **e**, Representative enrichment plots of mitochondrial fatty acid β oxidation and the TCA cycle/respiratory electron transport pathways in GS-treated mutant iMNs.

To investigate UBQLN2 function in ALS/FTD, we introduced X-linked UBQLN2 mutations (P497H or P506T) into a male human iPSC line using CRISPR-mediated gene editing. Whole-genome sequencing confirmed that the engineered iPSCs were nearly identical to their isogenic control, with normal karyotype, morphology, growth and pluripotency (Supplementary Fig. 1). We then differentiated these iPSCs into motor neurons (iMNs) carrying UBQLN2 mutations and profiled global protein turnover using stable isotope labeling by amino acids in cell culture (SILAC)-based quantitative mass spectrometry (Fig. 1a). Given the glucose hypometabolism and mitochondrial dysfunction reported in patients with ALS/FTD^1,2,9–15^, we assessed protein turnover in iMNs under both basal (complete medium (CM)) and metabolic (glucose starvation (GS)) stress conditions. Isogenic and mutant iMNs (P506T or P497H) were pulsed with heavy lysine and arginine, in the presence or absence of glucose. Protein half-lives were determined from heavy-to-light peptide abundance ratios. We quantified 6,940 and 6,326 proteins under CM and GS conditions, respectively, and observed that UBQLN2 mutations caused widespread alterations in protein turnover (Fig. 1b and Supplementary Table 1). Under CM, 3,331 of 6,940 (P506T) and 2,562 of 6,940 (P497H) proteins exhibited altered half-lives (Extended Data Fig. 1a), whereas, under GS, 2,025 of 6,326 (P506T) and 2,072 of 6,326 (P497H) proteins were affected (Extended Data Fig. 1b). Interestingly, UBQLN2 mutants demonstrated strong concordance in protein turnover profiles, with 2,170 proteins changing similarly in CM and 1,333 in GS (Fig. 1b,c). Most of the affected proteins showed slower turnover in the mutants across both conditions (Extended Data Fig. 1a,b), with 329 proteins uniquely altered under GS in both mutants (Fig. 1b), indicating that energy stress exacerbates proteostatic disturbances. These data demonstrate that ALS/FTD-linked UBQLN2 mutations globally prolong proteomic half-lives.

To identify biological pathways affected by altered global protein turnover, we performed gene set enrichment analysis (GSEA) using GOCC, GOMF, Hallmark and Reactome databases. Consistent with the role of UBQLN2 in protein degradation and stress granule dynamics, the enriched biological processes included protein quality control, RNA metabolism and translation (Fig. 1d and Supplementary Table 2). Parallel enrichment of carbohydrate metabolism, particularly glucose and lipid pathways, suggests hypermetabolism in UBQLN2 mutant iMNs (Fig. 1d and Extended Data Fig. 1c). In addition, GSEA revealed upregulation of cellular starvation responses, indicating a heightened sensitivity to energy deprivation in mutant iMNs (Extended Data Fig. 1c). These results suggest that dysregulated protein half-lives underlie metabolic dysfunction in UBQLN2 mutant iMNs, particularly under energy stress.

Given the distinct pattern of proteomic turnover between mutants and isogenic iMNs under GS (Extended Data Fig. 1d), we next examined the metabolic pathways most affected by UBQLN2 mutations. GSEA uncovered elevated mitochondrial functions alongside reduced neuronal functions in mutant iMNs under GS (Fig. 1e,f), with less pronounced changes under CM (Fig.1e). Lipids serve as an alternative energy source when glucose is limited^17,39^. We observed that mitochondrial fatty acid oxidation (FAO) was overactivated in mutant iMNs under GS (Fig. 1e,f and Extended Data Fig. 1e), potentially exacerbating metabolic imbalances under energy stress.

### Integrated omics uncover metabolic and neuronal deficits in UBQLN2 mutant neurons

**Fig. 2:**
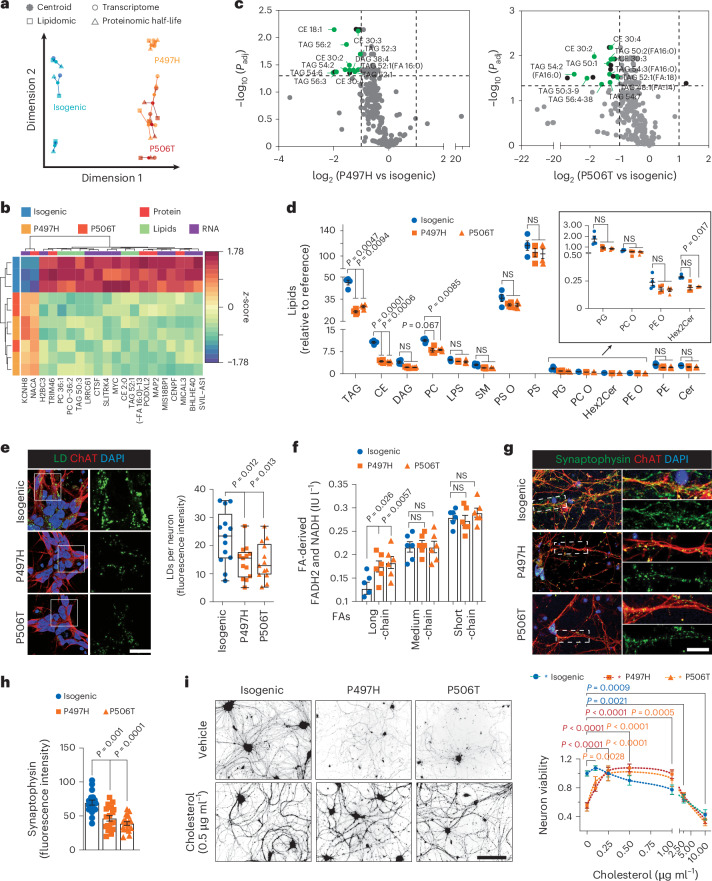
Multi-omic analyses identify metabolic changes in UBQLN2 mutant motor neurons. **a**, Integrated sparse partial least squares discriminant analysis (sPLS-DA) separates mutant and control motor neurons by combined lipidomic, transcriptomic and proteomic half-life profiles. Samples are color coded by genotype (blue, control; orange, mutant), and feature types are denoted by different symbols. **b**, Top-ranked features contributing to component 1 separation include dysregulated protein turnover, lipids and mRNA transcripts. Values were converted to *z*-scores. **c**, Volcano plots display significantly altered lipid species (*P*_adj_ < 0.05, fold change > 2) in mutant neurons, including reduced levels of major LD-associated species such as TAGs, DAGs and CEs (green). Multiple hypothesis testing corrections were applied using the Benjamini–Hochberg FDR procedure (two-sided). **d**, Lipidomic quantification shows the changes in 14 lipid species in UBQLN2 mutant iMNs compared to isogenic controls under GS. For each lipid species, statistical differences among the four groups (*n* = 4 biological replicates in two independent experiments per group) were assessed using the Brown–Forsythe and Welch ANOVA test with a post hoc analysis of Dunnett’s test (two-sided). Data are presented as mean ± s.e.m. **e**, Representative images and quantification of BODIPY-labeled LDs in iMNs after glucose deprivation for 24 hours. The average fluorescence intensity per field, with normalization to the number of neurons, was quantitatively analyzed (*n* = 13 fields of 92 neurons for isogenic, 97 neurons for P497H and 100 neurons for P506T; data were collected from three independent experiments). Scale bar, 10 μm. **f**, Measurement of mitochondrial β-oxidation activity in iMNs based on the production of FADH_2_ and NADH from long-chain, medium-chain and short-chain fatty acids. Isogenic control and mutant iMNs were cultured under GS for 24 hours (*n* = 5 biological replicates in three independent experiments). For **e** and **f**, group differences were assessed by ordinary one-way ANOVA with Dunnett’s multiple comparisons versus the control (two-sided). **g**,**h**, Representative immunofluorescence images (**g**) and quantification of synaptophysin fluorescence intensity (**h**) show reduced synaptophysin intensity in mutant iMNs under GS for 2 days (*n* = 18 fields in three independent experiments). Each dot in the graph represents the average fluorescence intensity of a field. Scale bar, 20 μm. Group differences were assessed by the Kruskal–Wallis test with Dunn’s post hoc test for multiple comparisons (two-sided). **i**, Calcein-AM staining shows reduced neuronal survival in UBQLN2 mutant iMNs under GS, which is mitigated by exogenous cholesterol supplementation in a dose-dependent manner (*n* = 6 biological replicates in two independent experiments). Scale bar, 100 μm. Two-way ANOVA followed by Tukey’s post hoc test was performed to assess the effects of genotype and doses (two-sided). For **f**, **h** and **i**, data are presented as mean ± s.e.m. For **e**, box plots are presented in the form of minima to maxima. FA, fatty acid; NS, not significant. Source data

**Extended Data Fig. 2:**
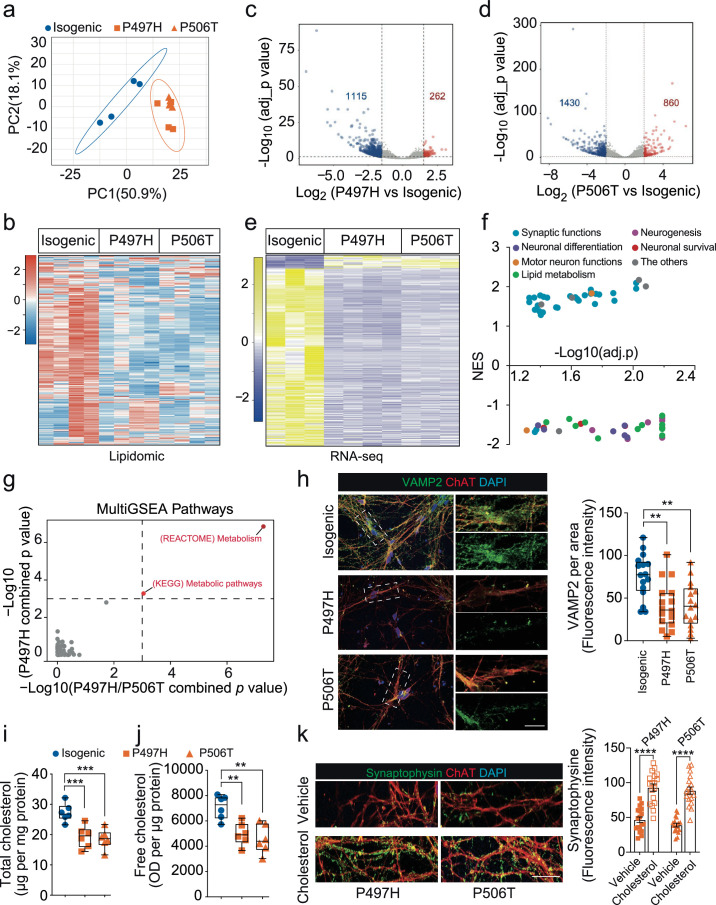
Molecular dysregulations in motor neurons harboring disease-linked UBQLN2 mutations. **a**, Principal component analysis (PCA) of lipidomic profiles comparing mutant UBQLN2 iMNs and isogenic controls following 24 hours of GS (n = four biological replicates in two independent experiments). **b**, Heatmap illustrating altered lipid species in mutant iMNs compared to controls under GS. Motor neurons were grown in glucose-free medium for 24 hours. Values were converted to Z-scores (n = four biological replicates in two independent experiments). **c** and **d**, Volcano plots of transcriptomic changes reveal predominant downregulation of genes in P497H (**c**) and P506T (**d**) mutant iMNs under glucose starvation (adj_p < 0.05, fold change > 2). **e**, Heatmap of differentially expressed genes under GS conditions. A total of 484 genes showed similar expression trends in both UBQLN2 mutant lines. Values were converted to Z-scores (n = three to four biological replicates). **f**, GSEA-based categorization of pathways altered in mutant iMNs under GS reveals dysfunctions in synaptic signaling, neuronal differentiation/survival, and lipid metabolism. **g**, Multi-omics gene set enrichment analysis (MultiGSEA) of proteomic half-lives, lipidomic, and transcriptomic data sets highlights convergent metabolic pathway dysregulation in both P497H and P506T mutant lines. Dots represent pathways based on their “p-values”. Pathways are labeled if the Bonferroni-adjusted p-value is < 0.05 in P497H or P506T. **h**, VAMP2 immunostaining highlights reduced synaptic vesicle density in P497H and P506T mutant iMNs under GS for two days. Quantification of VAMP2 fluorescence intensity per field across genotypes (n = 16 fields in three independent experiments). Scale bar, 10 μm. **i** and **j**, Quantification of total and free cholesterol demonstrates the loss of cholesterol in iMNs under GS for 24 hours (n = six biological repeats from three independent experiments). **k**, Cholesterol supplementation (0.25 μg/mL) elevated synaptophysin fluorescence intensity in UBQLN2 mutant iMNs under GS for two days. Quantification of synaptophysin signal per field (n = 19 fields in three independent experiments). Scale bar, 10 μm. For panels **h**-**k**, group differences were assessed by the Kruskal-Wallis test, respectively, with multiple comparisons controlled using Dunn’s test (two-sided) for **h**, **j**, and **k**, and the Benjamini-Krieger-Yekutieli two-stage step-up procedure (two-sided) for **i**. For panel **k**, data are presented as mean ± SEM. For panels **h**-**j**, box plots are presented as minima to maxima. For panel **k**, data are presented as mean ± SEM. Source data

Given the increased sensitivity of UBQLN2 mutant iMNs to energy stress, we conducted lipidomic and transcriptomic profiling of the mutant and isogenic iMNs under GS to comprehensively characterize metabolic alterations. Clustering analysis of the lipidomic data showed that UBQLN2 mutant iMNs exhibited similar lipid metabolic profiles, which were distinct from the isogenic control (Extended Data Fig. 2a,b and Supplementary Table 3). Specifically, both mutant iMNs showed a greater number of downregulated lipid species (Extended Data Fig. 2b). Transcriptomic analysis revealed widespread transcriptional repression in mutant iMNs under GS. The P497H mutation was associated with 1,115 downregulated genes and 262 upregulated genes, and the P506T mutation resulted in 1,430 downregulated genes and 860 upregulated genes (Extended Data Fig. 2c–e and Supplementary Table 4). Of these, 484 genes were dysregulated in both mutants, with the majority (450 genes) downregulated (Extended Data Fig. 2e). GSEA of the transcriptome indicated that pathways related to lipid metabolism, synaptic function, motor neuron function and neuronal survival were impaired in mutant iMNs under GS (Extended Data Fig. 2f). Integrated analyses of proteomic half-lives, lipidomic profiles and transcriptomic data showed clear segregation between mutant and isogenic iMNs (Fig. 2a), with most variance captured along the first principal component (Fig. 2b). Multi-GSEA across the three omics layers identified metabolism as the most significantly enriched biological process in both UBQLN2 mutant iMNs (Extended Data Fig. 2g), underscoring a critical role of UBQLN2 in metabolic regulation, a process dysregulated in ALS/FTD.

### UBQLN2 mutations impair lipid metabolism and synaptic vesicle integrity

LDs are dynamic organelles that store neutral lipids and serve to support energy metabolism and membrane functions^39,40^. Lipidomic analysis revealed significant reductions in LD-associated lipids, particularly cholesterol esters (CEs) and triacylglycerols (TAGs), in UBQLN2 mutant iMNs (Fig. 2c,d), which were validated by BODIPY staining (Fig. 2e). Because LDs fuel mitochondrial β-oxidation^17,20,39^, we examined whether LD depletion was associated with increased FAO under GS by measuring FADH_2_ and NADH levels produced from fatty acids of varying chain lengths in UBQLN2 mutant and isogenic control iMNs. We observed no difference in FADH_2_ or NADH levels generated from short-chain or medium-chain substrates (butyryl-CoA and octanoyl-CoA) between mutant and control iMNs. However, FADH_2_ and NADH levels from the long-chain substrate (palmitoyl-CoA) were significantly higher in UBQLN2 mutant iMNs compared to isogenic controls (Fig. 2f), indicating overactivation of long-chain FAO under stress. This observation is consistent with the GESA of proteomic half-lives (Extended Data Fig. 1e) and the reduced abundance of long-chain fatty-acid-containing TAGs in lipidomic profiles (Fig. 2c).

Multi-omic analyses indicated notable dysregulation of neuronal functions, particularly in synapse-related pathways (Fig. 1f). Immunostaining of synaptic vesicle markers VAMP2 and synaptophysin^41,42^ in UBQLN2 mutant iMNs indicated compromised synaptic vesicle integrity, as reflected by reduced fluorescence intensities of these markers after 2 days of GS (Fig. 2g,h and Extended Data Fig. 2h). Prolonged GS (3 days) decreased the viability of mutant iMNs relative to controls (Fig. 2i). Given the essential role of cholesterol in maintaining membrane curvature, vesicle stability and protein microdomains^42–44^, we asked whether cholesterol loss contributes to these phenotypes. Under GS, both total and free cholesterol were significantly reduced in UBQLN2 mutant iMNs (Extended Data Fig. 2i,j), consistent with the observed synaptic vesicle deficits. Cholesterol supplementation produced a biphasic response: a high dose (10 μg ml^−1^) worsened viability in both UBQLN2 mutant and control iMNs, whereas lower doses (0.1–1 μg ml^−1^) selectively improved viability in mutants after 3 days of GS (Fig. 2i). Notably, 0.25 μg ml^−1^ cholesterol robustly restored synaptic vesicle levels in mutant iMNs (Extended Data Fig. 2k). This neuroprotective effect of cholesterol on viability was absent in isogenic control iMNs (Fig. 2i), suggesting that cholesterol deficiency contributes specifically to the vulnerability of UBQLN2 mutant iMNs. Together, these observations suggest that lipid dysregulation, particularly LD depletion, overactivated FAO and cholesterol deficiency, collectively contribute to neurodegeneration in UBQLN2-linked ALS/FTD.

### UBQLN2 mutant brain organoids exhibit LD deficits and neuronal apoptosis

**Fig. 3:**
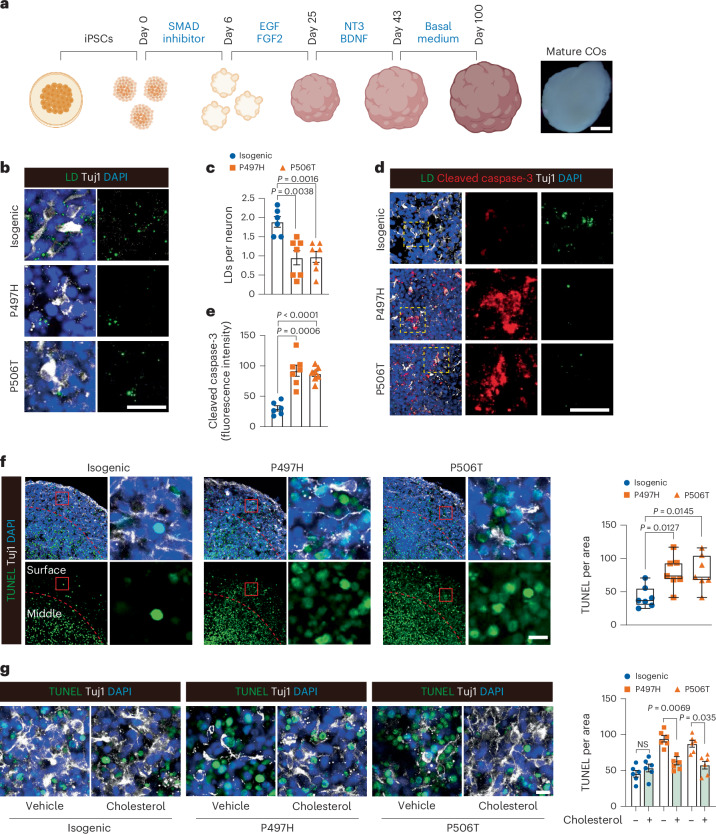
Brain organoids with UBQLN2 mutations exhibit lipid dysregulation and neurodegenerative phenotypes under energy stress. **a**, Schematic timeline outlining the differentiation of human iPSC-derived COs, including dual-SMAD inhibition and growth factor patterning, followed by long-term maturation to day 100. **b**–**e**, Representative images and quantification of BODIPY-labeled LDs (**b**,**c**), cleaved caspase-3 (**d**,**e**) and Tuj1-positive neurons in isogenic control and UBQLN2 mutant COs under GS. On day 100, mature COs were subjected to 3 days of GS. Six fields in each CO were examined. Each dot in the graphs represents the average fluorescence intensity of LDs or cleaved caspase-3 per neuron in an organoid (for **c** and **e**, *n* = 6 for isogenic and *n* = 7 for P497H/P506T). Scale bar, 10 μm. **f**, TUNEL and Tuj1 staining show increased neuronal apoptosis in the surface regions of UBQLN2 mutant COs after GS for 3 days. The average fluorescence intensity of TUNEL in an organoid was statistically analyzed (*n* = 6 for isogenic and *n* = 7 for P497H/P506T). Scale bar, 10 μm. **g**, Cholesterol supplementation (1 μg ml^−1^) reduced neuronal apoptosis in UBQLN2 mutant COs under GS. Neuronal apoptosis in the surface regions of COs was examined by TUNEL and Tuj1 staining after 3 days of GS with or without cholesterol supplementation (1 μg ml^−1^). Each dot in the graphs represents the average fluorescence intensity of TUNEL staining in an organoid (*n* = 6 for isogenic and *n* = 7 for P497H/P506T). Scale bar, 10 μm. For **c** and **e**–**g**, group differences were assessed by Brown–Forsythe and Welch ANOVA test with a post hoc analysis of Dunnett’s test (two-sided). For **c**, **e** and **g**, data are presented as mean ± s.e.m. For **f**, the box plots are presented in the form of minima to maxima. Schematic in **a** created in BioRender; Wang, J. https://biorender.com/tscxymp (2025). Source data

**Extended Data Fig. 3:**
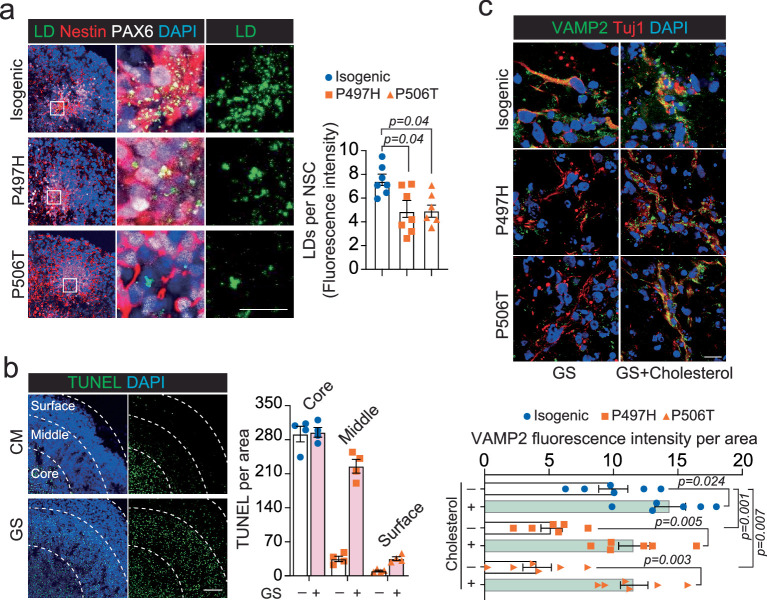
Deficiencies of LDs and synaptic vesicles in UBQLN2 mutant cortical organoids. **a**, Reduced LD content in the neurogenesis clusters of UBQLN2 mutant COs. On day 25 of differentiation, LDs were stained by BODPY(493/503) alongside neural stem cell (NSC) markers Nestin and PAX6. Six fields in each cortical organoid were examined. Each dot represents the average LD fluorescence per NSC in an organoid (n = 7 for isogenic, n = 7 for P497H, and n = 6 for P506T). Scale bar, 10 μm. **b**, TUNEL staining reveals elevated apoptosis in the middle zone of mature UBQLN2 mutant COs (day 100) following 3 days of glucose starvation (GS), compared to complete medium (CM). Each dot represents the average TUNEL signal in a region per organoid (n = four COs). Scale bar, 200 μm. **c**, Representative images of synaptic vesicle marker VAMP2 in mature COs with or without 1 μg/mL cholesterol supplementation during GS. Synaptic vesicle density was reduced in mutant iMNs but increased following cholesterol supplementation (n = 6 COs). Scale bar, 20 μm. For panels **a**, group differences were assessed by the Kruskal-Wallis test, with multiple comparisons controlled using Dunn’s test (two-sided). For panel **c**, group differences were assessed by Brown-Forsythe and Welch ANOVA test with Benjamini-Krieger-Yekutieli two-stage step-up procedure (two-sided). Data are presented as mean ± SEM for panels **a**-**c**. Source data

To examine metabolic phenotypes of ALS/FTD-linked UBQLN2 in a three-dimensional model, we generated cerebral cortical organoids (COs) from iPSCs using a previously described protocol (Fig. 3a)^45^. We first assessed LDs at day 25, a stage characterized by rapid cellular expansion and high energy expenditure. In isogenic COs, LDs were abundant in central neurogenesis clusters enriched for Nestin/PAX6-positive neurons (Extended Data Fig. 3a). By contrast, UBQLN2 mutant COs exhibited significant LD deficits in these regions (Extended Data Fig. 3a), indicating increased susceptibility to LD dysregulation. We next assessed LD metabolism and neuronal integrity in mature COs under GS. After 100 days of differentiation, COs were incubated in glucose-free medium for 3 days, followed by quantification of neuronal LDs and cleaved caspase-3 levels. Compared to isogenic controls, mutant COs showed markedly reduced LDs and increased cleaved caspase-3 staining in Tuj1-positive neurons (Fig. 3b–e), indicating impaired lipid metabolism and associated neuronal apoptosis in this mini-brain model of UBQLN2-linked ALS/FTD.

To spatially analyze neuronal vulnerability under metabolic stress, we quantified apoptosis and synaptic vesicle abundance across core, middle and surface regions. Under basal conditions, TUNEL-positive cells were primarily localized to the core (Extended Data Fig. 3b). After 3 days of GS, apoptotic cells extended into the middle zone, with relatively few events at the surface (Extended Data Fig. 3b), suggesting that the surface region is most sensitive for detecting stress-induced differences. Indeed, mutant COs exhibited significantly more apoptotic neurons and fewer synaptic vesicles than controls in the surface regions (Fig. 3f and Extended Data Fig. 3c). Cholesterol supplementation (1 μg ml^−1^) during GS reduced apoptosis and restored synaptic vesicle levels in mutant COs (Fig. 3g and Extended Data Fig. 3c). Thus, UBQLN2 mutant organoids exhibit heightened vulnerability to metabolic stress, which can be mitigated by cholesterol supplementation.

### UBQLN2 regulates intracellular lipid homeostasis

**Fig. 4:**
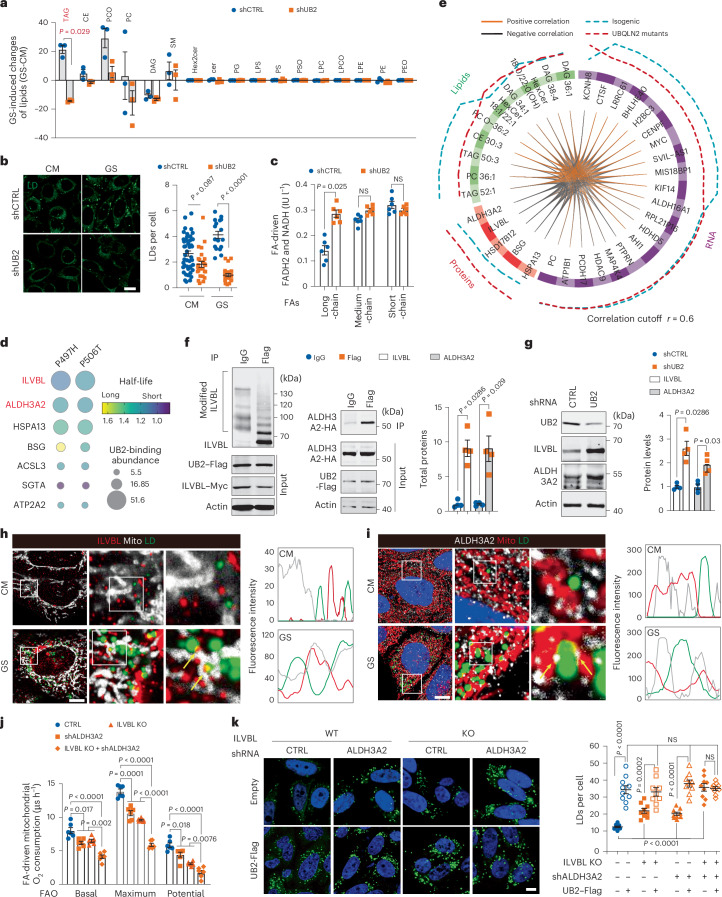
UBQLN2 regulates lipid homeostasis by targeting ILVBL and ALDH3A2 for proteasomal degradation. **a**, Lipidomic profiling revealed selective loss of TAG in UBQLN2-depleted HeLa cells under GS. HeLa cells with or without UBQLN2 knockdown were starved with glucose-free medium for 24 hours (*n* = 3 biological replicates in three independent experiments). **b**, BODIPY(493/503) staining shows increased LD accumulation in shUBQLN2 cells cultured in CM or GS for 24 hours. Quantification shows the number of LDs per cell across multiple fields (for CM, *n* = 46 fields of 1,726 cells for shCTRL and *n* = 25 fields of 792 cells; for GS, *n* = 17 fields of 458 cells for shCTRL and *n* = 22 fields of 527 cells; data were collected from three independent experiments). Each dot in the graph represents the average number of LDs in a field normalized against cell number. Scale bar, 10 μm. **c**, Mitochondrial FAO capacity was assessed by FADH_2_ and NADH production in shCTRL and shUBQLN2 HeLa cells under GS with the supplementation of long-chain, medium-chain and short-chain fatty acids (*n* = 6 biological replicates). **d**, Integrated proteomic and interactome analyses identify ILVBL and ALDH3A2 among UBQLN2 clients with altered protein half-lives in UBQLN2 mutant motor neurons specifically under GS. **e**, The circos plot visualizes multi-omics correlations (*r* > 0.6) among dysregulated turnover of UBQLN2’s client proteins, transcripts and lipids in isogenic and UBQLN2 mutant iMNs under GS. **f**, Co-immunoprecipitation confirms UBQLN2’s interaction with ILVBL and ALDH3A2 in HeLa cells. HeLa cells were transfected with UBQLN2–Flag and ILVBL–Myc or ALDH3A2–HA for 24 hours, followed by immunoprecipitation using IgG or anti-Flag (*n* = 2 biological replicates in two independent experiments). **g**, Immunoblotting analyses demonstrate elevated ILVBL and ALDH3A2 protein levels after UBQLN2 knockdown (*n* = 2 biological replicates in two independent experiments). **h**,**i**, Immunostaining reveals that ILVBL (**h**) and ALDH3A2 (**i**) are enriched at LD and mitochondria including their contact sites (arrows), particularly under GS. HeLa cells were starved with glucose-free medium for 24 hours (*n* = 12 fields from three independent experiments). Scale bar, 5 μm. **j**, Mitochondrial oxygen consumption driven by 2-hydroxy-oleic acid was reduced upon ILVBL and/or ALDH3A2 depletion under GS for 24 hours, indicating impaired FAO. The basal, maximum and potential capacity of mitochondrial O_2_ consumption was measured and analyzed (*n* = 6 biological replicates). **k**, LD staining and quantification in HeLa cells, with or without shALDH3A2, ILVBL KO or ectopic expression of UBQLN2, under GS. Cells were transfected with UBQLN2 for 24 hours, followed by an additional 24 hours of GS (*n* = 9–10 fields of 89–103 cells in three independent experiments; scale bar, 10 μm). Statistical analyses for **a** and **b** were performed using Brown–Forsythe and Welch ANOVA test with a post hoc analysis of Dunnett’s test (two-sided). For **c** and **k**, group differences were assessed by the Kruskal–Wallis test, with a post hoc analysis of Dunnett’s test (two-sided) and Benjamini–Krieger–Yekutieli two-stage step-up procedure (two-sided), respectively. For **f** and **g**, statistical differences were assessed using the Mann–Whitney *U*-test (two-sided). For **j**, group differences were assessed by Brown–Forsythe and Welch ANOVA test with Benjamini–Krieger–Yekutieli two-stage step-up procedure (two-sided). **i** was analyzed using an unpaired two-tailed Student’s *t*-test. For **k**, group differences were assessed using one-way Welch ANOVA with Games–Howell post hoc. All statistical tests were two-sided. Data are presented as mean ± s.e.m. for **a**–**c**, **f**, **g**, **j** and **k**. CTRL, control; FA, fatty acid; IP, immunoprecipitation; KO, knockout; NS, not significant. Source data

**Extended Data Fig. 4:**
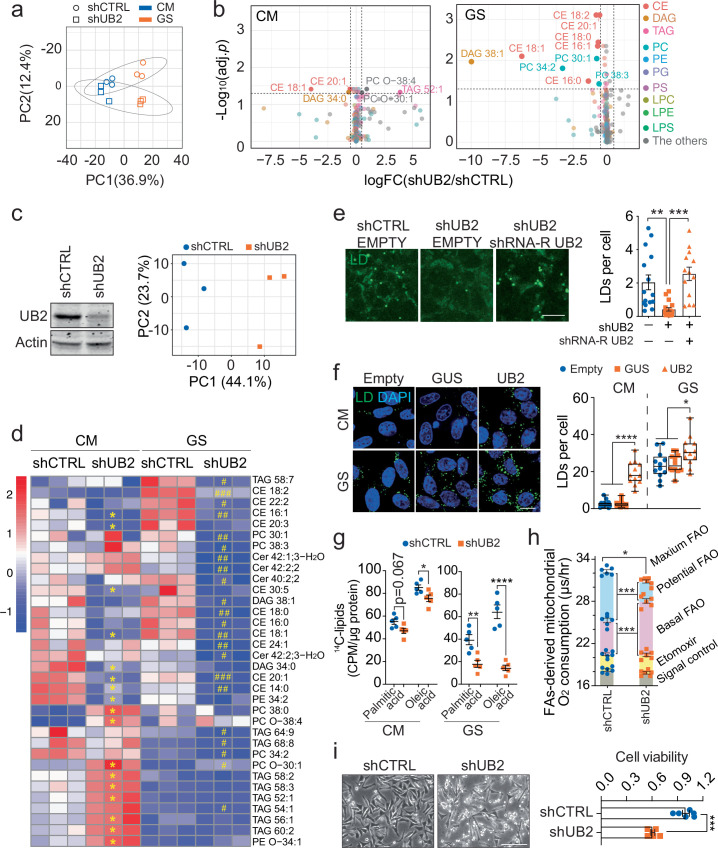
UBQLN2 regulates intracellular lipid droplet homeostasis and fatty acid metabolism. **a**, Principal component analysis (PCA) of the lipidomic profiles in HeLa cells transfected with control or UBQLN2-targeting shRNAs, followed by 24 hours of GS or CM culture (n = three biological replicates). **b**, Volcano plot showing the loss of lipid species in shUBQLN2 HeLa cells under CM or GS for 24 hours (adj_p < 0.05, fold change > 1.5). **c**, Immunoblotting images displaying the knockdown efficiency of UBQLN2 shRNA in HEK293 cells (left panel). PCA (right panel) of lipidomic datasets in UBQLN2-depleted HEK293 cells after 24 hours of GS (n = three biological replicates in three independent experiments). **d**, Heatmap displaying the lipid species significantly (adj_p < 0.05) altered by UBQLN2 knockdown under CM or GS for 24 hours in HeLa cells. Values were converted to Z-scores (n = three biological replicates in three independent experiments; shCTRL vs shUBQLN2 under CM: *p < 0.05; shCTRL vs shUBQLN2 under GS: # p < 0.05, ## p < 0.01, ### p < 0.001). **e**, Quantification of LDs using BODIPY(493/503) staining in HEK293 cells under GS, with or without re-expression of a shRNA-resistant UBQLN2 construct (n = 15 fields of 398 cells for shCTRL, n = 18 fileds of 441 cells for shUB2, and n = 13 fields of 272 cells for shUB2+shUB2-R UB2. Data were collected from three independent experiments). Each dot represents the average number of LDs in a field, normalized against cell number. Scale bar, 10 μm. **f**, Ectopic expression of UBQLN2 reduced LD accumulation in HeLa cells cultured in CM or under GS (n = 12 fields of 86 cells for CM+empty, 82 cells for CM + GUS, 81 cells for CM + UB2, 80 cells for GS+empty, 82 cells for GS + GUS, and 76 cells for GS + UB2. Data were collected from three independent experiments). Each dot in the graphs represents the average number of LDs in a field. Scale bar, 10 μm. **g**, UBQLN2 knockdown impaired degradation of 14C-palmitate and 14C-oleate under GS. shCTRL and shUBQLN2 HeLa cells were cultured in glucose-free medium for 24 hours and fed with 14C-labeled oleic or palmitic acids (n = five biological replicates in five independent experiments). **h**, Measurement of fatty acid-driven oxygen consumption shows the compromised mitochondrial capacity of catabolizing FAs in HeLa cells upon the loss of UBQLN2 under GS. HeLa cells were transfected with control or UBQLN2-targeting shRNA for 24 hours followed by another 24 hours of GS. Maximum, basal, and potential capacity of mitochondrial generation of O2 were analyzed (n = six biological replicates in each group). **i**, Crystal violet staining shows reduced survival of UBQLN2-knockdown (shUBQLN2) HeLa cells after 48 hours of glucose starvation (GS) compared to control (shCTRL) (n = six biological replicates in three independent experiments). Scale bar, 100 μm. For panel **e**, statistical significance was assessed by Kruskal-Wallis test, with multiple comparisons controlled using a post-hoc analysis of Dunnett’s test (two-sided). For panel **f**, group differences were assessed by ordinary one-way ANOVA with Sidak test for the correction of multiple comparisons. For **g** and **h**, group differences were assessed by Kruskal-Wallis test, respectively, with multiple comparisons controlled using a post-hoc analysis of Benjamini-Krieger-Yekutieli two-stage step-up procedure (two-sided) and Dunnett’s test (two-sided). For panel **i**, statistical analysis was performed using an unpaired Mann-Whitney U test (two-sided). For panels **e** and **g**-**i**, data are presented as mean ± SEM. For panel **f**, box plots are presented as minima to maxima. Source data

**Extended Data Fig. 5:**
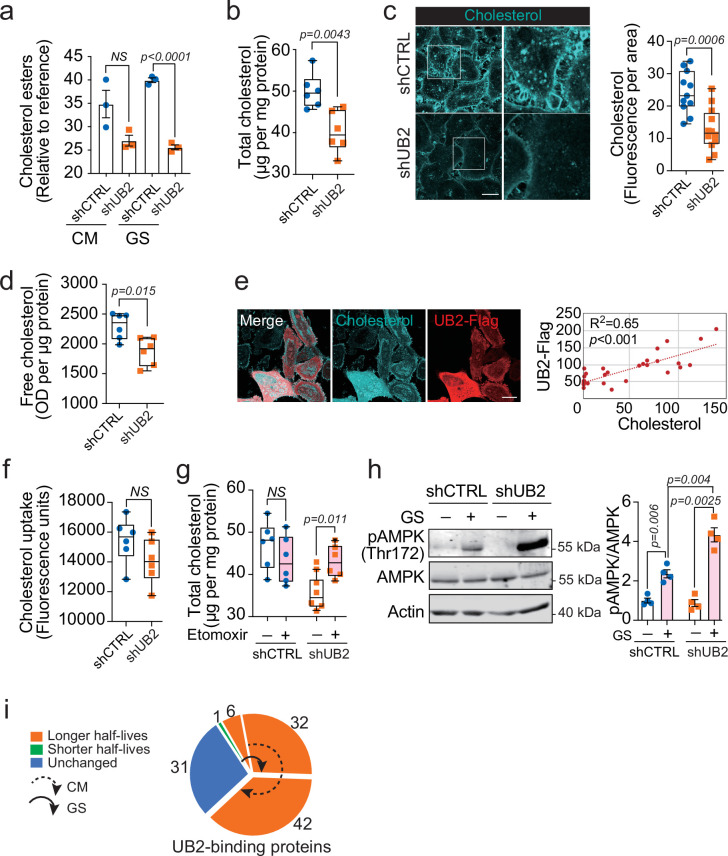
UBQLN2 regulates intracellular cholesterol synthesis. **a**, Lipidomic analyses show reduced levels of cholesterol esters (CEs) in HeLa cells with UBQLN2 knockdown, particularly under GS conditions (n = three biological replicates in three independent experiments). **b**-**d**, Measurements of cholesterol demonstrate the loss of both total (**b**) and free cholesterol (**c**,**d**) in shUBQLN2 cells under 24 hours of GS. Free cholesterol was measured by Filipin III staining (**c**), which was further confirmed by a colorimetric assay (**d**) (**a**, **b** and **d**, n = six biological replicates in three independent experiments; **c**, n = 11 fields for shCTRL and n = 13 fields for shUB2. Data were collected from three independent experiments. Each dot represents the average number of fluorescence intensity in a field). Scale bar, 10 μm. **e**, Filipin III staining of HeLa cells expressing UBQLN2-Flag shows a positive correlation between UBQLN2 expression and intracellular cholesterol levels (n = 29 fields in three independent experiments). Each dot in the graphs represents the average fluorescence intensity in a field. Scale bar, 10 μm. **f**, Cholesterol uptake capacity was unchanged in UBQLN2-knockdown cells compared to controls after 24 hours GS (n = six biological replicates from two independent experiments). **g**, Etomoxir treatment mitigated cholesterol loss in shUBQLN2 cells under GS (10 µM, 24 hours), suggesting cholesterol biosynthesis was associated with FAO (n = six biological replicates from two independent experiments). **h**, Immunoblotting analyses show elevated phosphorylation of AMPK (Thr172) in shUBQLN2 cells under GS for 6 hours (n = four biological replicates from two independent experiments). **i**, Integrated proteomic and interactome analysis revealed that six UBQLN2-interacting proteins exhibit extended half-lives selectively under GS in mutant iMNs. Brown-Forsythe and Welch ANOVA test with a post-hoc analysis of Dunnett’s test (two-sided) were applied for the statistical analysis for panel **a**. For panels **b**-**d**, and **f**, statistical analyses were performed using an unpaired Mann-Whitney U test (two-sided). For panels **g** and **h**, group differences were assessed by the Brown-Forsythe and Welch ANOVA test with a post-hoc analysis of Dunnett’s test (two-sided). For panels **a** and **h**, data are presented as mean ± SEM. For panels **b**-**d**, **f**, and **g**, box plots are presented as minima to maxima. Source data

To define the role of UBQLN2 in lipid metabolism, we performed lipidomic profiling of UBQLN2 knockdown HeLa cells under both GS and basal CM conditions. A total of 430 lipids across 17 classes were quantified (Supplementary Table 5). Principal component analysis showed minimal differences at baseline but a marked divergence under GS (Extended Data Fig. 4a,b). Similar GS-dependent alterations were observed in UBQLN2 knockdown HEK293 cells (Extended Data Fig. 4c and Supplementary Table 6), indicating that UBQLN2 loss perturbs lipid homeostasis under metabolic stress. Notably, under GS, UBQLN2 knockdown led to pronounced reductions in TAGs and CEs compared to controls (Fig. 4a and Extended Data Figs. 4b,d and 5a). Because TAGs and CEs are major components of LDs^39,40^, we examined LD dynamics and found that UBQLN2 knockdown modestly reduced LDs at baseline but markedly depleted LDs under GS (Fig. 4b), consistent with observations in UBQLN2 mutant iMNs (Fig. 2f). This LD deficiency phenotype was absent in HEK293 cells expressing short hairpin RNA (shRNA)-resistant UBQLN2 (Extended Data Fig. 4e). Conversely, UBQLN2 overexpression, but not that of the control protein β-glucuronidase (GUS), increased LD abundance under both basal and GS conditions (Extended Data Fig. 4f), demonstrating that UBQLN2 is required for LD homeostasis.

Given the elevated FAO and respiration in UBQLN2 mutant iMNs under GS, we asked whether UBQLN2 modulates mitochondrial lipid catabolism. Using ^14^C-labeled palmitate and oleate—long-chain fatty acids stored as TAG and DAG in LDs^39^—we observed that UBQLN2 knockdown accelerated lipid breakdown, especially under GS (Extended Data Fig. 4g). Measurement of fattty-acid-derived FADH_2_ and NADH showed that UBQLN2 loss selectively increased the oxidation of long-chain fatty acids but not of medium-chain fatty acids or short-chain fatty acids (Fig. 4c). Consistently, basal FAO was elevated, whereas maximal and spare FAO capacities were reduced (Extended Data Fig. 4h), indicating reduced metabolic flexibility. Moreover, cell viability declined after 2 days of GS (Extended Data Fig. 4i). Hence, UBQLN2 regulates mitochondrial lipid catabolism to support cellular adaptability under metabolic stress.

We next analyzed cholesterol homeostasis, which underpins membrane function and synaptic vesicle biogenesis^46^. Lipidomic analyses showed significant CE reductions upon UBQLN2 knockdown in HeLa cells (Extended Data Fig. 5a). Consistent with CE deficiency, total and free cholesterol levels declined under GS (Extended Data Fig. 5b–d), whereas UBQLN2 overexpression increased cellular cholesterol in a dose-dependent manner (Extended Data Fig. 5e). Because cholesterol uptake was unchanged under GS (Extended Data Fig. 5f), these results suggest that the cholesterol deficit arises from impaired synthesis rather than reduced uptake.

Long-chain fatty acyl-CoA, an FAO intermediate, activates AMPK, which suppresses cholesterol synthesis^47,48^. Because UBQLN2 knockdown enhances mitochondrial FAO (Fig. 4c), we tested whether the enhanced mitochondrial FAO contributes to cholesterol loss. Treatment with etomoxir (10 µM), an inhibitor of carnitine palmitoyltransferase I that blocks long-chain fatty acid transport into mitochondria, increased cholesterol levels in UBQLN2-deficient cells but not control cells under GS (Extended Data Fig. 5g). Consistently, AMPK phosphorylation at Thr172 was elevated in UBQLN2 knockdown HeLa cells under GS (Extended Data Fig. 5h). Moreover, UBQLN2 interacted with acyl-CoA synthetase long chain family member 3 (ACSL3), an enzyme that generates long-chain fatty acyl-CoA^49^, and ACSL3 half-life was prolonged in UBQLN2 mutant iMNs under GS (Fig. 4d). Together, these data suggest that cholesterol depletion in UBQLN2-deficient cells results from enhanced mitochondrial FAO and subsequent AMPK activation.

### ILVBL and ALDH3A2 are key client enzymes of UBQLN2

To investigate how UBQLN2 regulates metabolism, we cross-referenced the UBQLN2 interactome with the proteomic turnover data in iMNs. Of 113 UBQLN2 interactors^30^, 22 were metabolic enzymes (Supplementary Table 7). Notably, 81 out of the 113 proteins displayed prolonged half-lives in UBQLN2 mutant iMNs compared to isogenic controls, whereas only one protein showed accelerated turnover. Among the 81 proteins with extended half-lives, 32 were altered under both CM and GS conditions, 42 under only CM and six exclusively under GS (Extended Data Fig. 5i).

**Extended Data Fig. 6:**
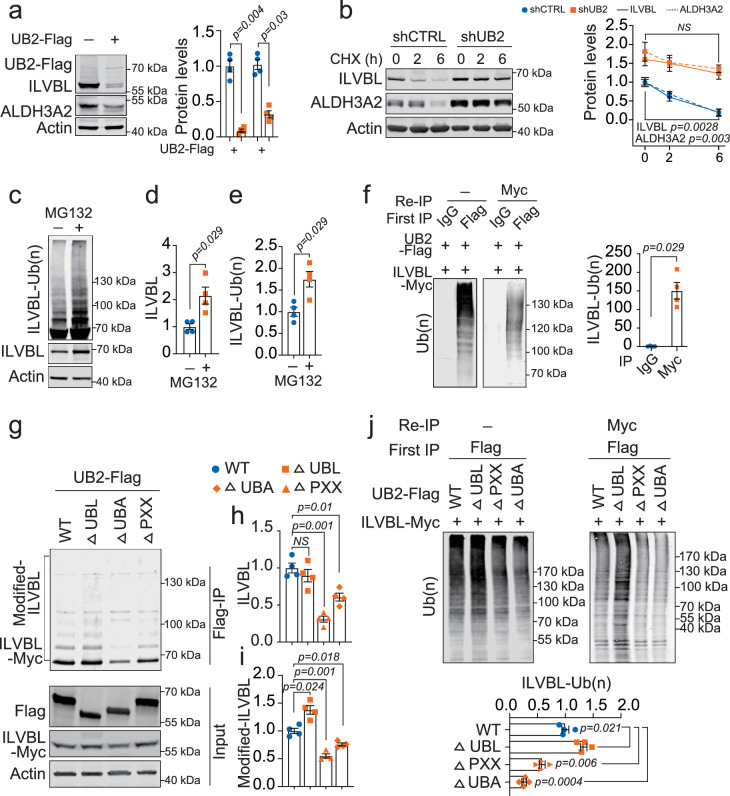
UBQLN2 mediates the ubiquitin-proteasomal degradation of ILVBL and ALDH3A2. **a**, Immunoblotting analyses demonstrate decreased levels of ALDH3A2 and ILVBL in HeLa cells upon ectopic expression of UBQLN2 (n = four biological replicates in four independent experiments). **b**, Cycloheximide (CHX) chase analysis reveals extended half-life of ALDH3A2 and ILVBL in shUBQLN2 HeLa cells under GS. Cells were treated with CHX (10 µg/mL) for 6 hours (n = four biological replicates in four independent experiments). **c**-**e**, Proteasome inhibition with MG132 increased ILVBL protein levels and polyubiquitinated ILVBL. HeLa cells were treated with MG132 at 10 μM for 8 hours (n = four biological replicates in four independent experiments). **f**, Co-immunoprecipitation assays demonstrate the interactions between UBQLN2 with polyubiquitinated ILVBL. HeLa cells were transfected with UBQLN2-Flag and ILVBL-Myc for 24 hours and subjected to the first immunoprecipitation (IP) using IgG or anti-Flag, followed by re-immunoprecipitation (Re-IP) using anti-Myc antibodies (n = four biological replicates in four independent experiments). **g**-**j**, The interaction of wild-type UBQLN2 and truncated mutants to ILVBL (**g**-**i**) and ubiquitinated ILVBL (**j**). HeLa cells were transfected with UBQLN2-Flag and ILVBL-Myc for 24 hours, followed by treatment with MG132 at 10 μM for 8 hours. Cells were subjected to immunoprecipitation (IP) using anti-Flag antibodies to pull down UBQLN2 and associated proteins (**g**). The precipitates from the first pull-down were then subjected to re-immunoprecipitation (Re-IP) using anti-Myc antibodies to isolate ILVBL proteins, followed by anti-poly-ubiquitin [Ub(n)] immunoblotting analysis (**j**) (n = four biological replicates in four independent experiments). For panel a, group differences were assessed by Kruskal-Wallis test, with multiple comparisons controlled using the Benjamini-Krieger-Yekutieli two-stage step-up procedure (two-sided). For panels **b** and **h**-**j**, group differences were assessed by Brown-Forsythe and Welch ANOVA test with a post-hoc analysis of Dunnett’s test (two-sided). For panels **d**-**f**, statistical significance was assessed by an unpaired Mann–Whitney U test (two-sided). Data are presented as mean ± SEM for panels **a**, **b**, **d**-**f**, and **h**-**j**. Source data

To identify UBQLN2 client proteins functionally relevant to metabolic dysregulation in mutant iMNs, we selected candidates with high binding affinity to UBQLN2 (≥10-fold enrichment over control; Supplementary Table 7) and altered turnover. These potential UBQLN2 client proteins were integrated with lipidomic and transcriptomic datasets for multi-omic correlation analyses. Using a correlation threshold of 0.6, five proteins—ILVBL, ALDH3A2, HSD17B12, BSG and HSPA13—exhibited the strongest associations with altered lipid species (for example, DAGs, TAGs and CEs) and specific transcripts (for example, MYC and CENPF) (Fig. 4e). These changes in lipids and transcripts were negatively correlated with the five proteins, all of which exhibited longer half-lives in mutant iMNs under GS (Fig. 4e). Among them, ILVBL and ALDH3A2 were the most enriched proteins identified in the UBQLN2 pulldown interactome (Supplementary Table 7). Both ILVBL and ALDH3A2 are key enzymes in lipid metabolism. ILVBL converts branched-chain fatty acids into fatty acyl-CoA esters (FA-CHO), and ALDH3A2 oxidizes FA-CHO to fatty acids that subsequently undergo β-oxidation^50^. Given the role of UBQLN2 in protein degradation^51^, we hypothesized that UBQLN2 maintains lipid homeostasis by regulating ILVBL and ALDH3A2 turnover. Interactions between UBQLN2 and either ILVBL or ALDH3A2 were validated by co-immunoprecipitation (Fig. 4f). Moreover, UBQLN2 modulated the turnover of both proteins, as evidenced by their increased abundance and extended half-lives in UBQLN2 knockdown cells and their reduced levels after UBQLN2 overexpression (Fig. 4g and Extended Data Fig. 6a,b).

We further dissected the mechanism by which UBQLN2 regulates the turnover of its client proteins using ILVBL as a model. ILVBL degradation was proteasome dependent, as treatment with MG132 induced ILVBL accumulation and increased its ubiquitination (Extended Data Fig. 6c–e). UBQLN2 co-immunoprecipitated with both unmodified and polyubiquitinated ILVBL (Fig. 4f), and sequential immunoprecipitation confirmed the presence of ubiquitinated ILVBL in the complex (Extended Data Fig. 6f). Domain mapping analyses showed that deletion of the UBA domain disrupted binding to both forms of ILVBL; UBL deletion enhanced binding to ubiquitinated ILVBL without affecting unmodified ILVBL; and PXX deletion impaired both interactions (Extended Data Fig. 6g–j). Thus, UBQLN2 engages ILVBL through distinct domains to promote its proteasomal degradation.

### UBQLN2 maintains lipid homeostasis through ILVBL and ALDH3A2

ILVBL and ALDH3A2, previously reported to localize to the endoplasmic reticulum and peroxisomes^50^, are also associated with LDs and mitochondria under energy stress (Fig. 4h,i). Under basal CM conditions, ILVBL showed minimal co-localization with LDs or mitochondria; however, under GS, it accumulated at both organelles and at LD–mitochondria contact sites (Fig. 4h). ALDH3A2 was already co-localized with mitochondria under CM (Fig. 4i) and, upon GS stress that increases LD abundance, redistributed to LD–mitochondria contact sites (Fig. 4i). These spatial dynamics suggest that both enzymes coordinate LD–mitochondria lipid metabolism during metabolic stress.

**Extended Data Fig. 7:**
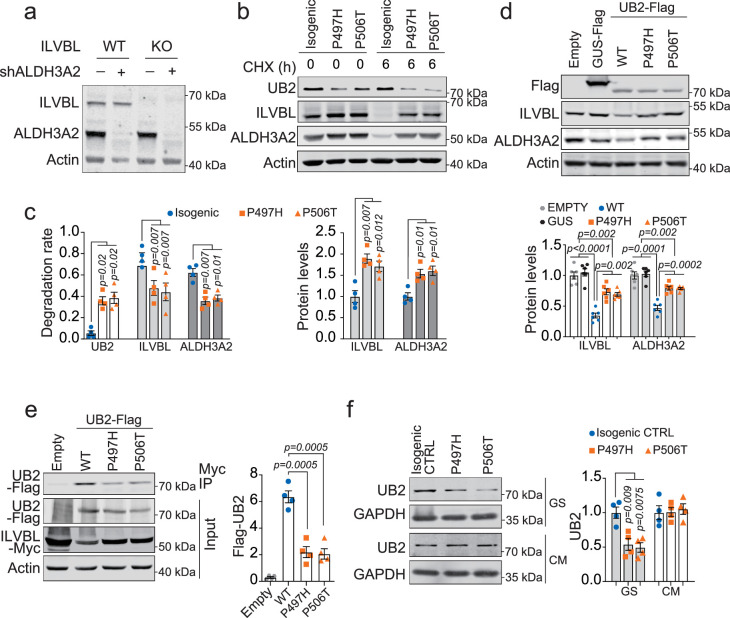
Dysregulated turnover of UBQLN2, ILVBL, and ALDH3A2 due to disease-linked UBQLN2 mutations. **a**, Immunoblot validation of ILVBL knockout (KO) and ALDH3A2 knockdown in HeLa cells. The experiments were repeated twice with two independent gRNAs and shRNAs, respectively, targeting ILVBL and ALDH3A2. **b** and **c**, Delayed degradation of ILVBL and ALDH3A2 in P497H and P506T neurons under GS. Isogenic and UBQLN2 mutant iMNs were starved with glucose-free medium containing CHX at 10 µg/mL for 6 hours (n = four biological replicates in four independent experiments). **d**, Immunoblotting analyses of ALDH3A2 and ILVBL protein levels in HeLa cells transfected with vector only, a control protein β-glucuronidase (GUS), WT, or mutant UBQLN2 for 24 hours (n = three biological replicates in two independent experiments). **e**, Co-immunoprecipitation analysis of the interaction of wild-type or mutant UBQLN2 with ILVBL. HeLa cells expressing ILVBL-Myc were transfected with the empty vector, wild-type, P497H, or P506T UBQLN2 for 24 hours, followed by a pull-down assay using anti-Myc (n = two biological replicates in two independent experiments). **f**, Decreased UBQLN2 protein levels in motor neurons differentiated from iPSCs carrying UBQLN2 (P497H or P506T) under GS. The UBQLN2 protein levels in motor neurons differentiated from isogenic or UBQLN2 mutant iPSCs cultured in CM or under GS for 24 hours were measured by immunoblotting (n = two biological replicates in two independent experiments). Group differences were all assessed by Brown-Forsythe and Welch ANOVA test with Benjamini-Krieger-Yekutieli two-stage step-up procedure (two-sided). Data are presented as mean ± SEM for panels **c**-**f**. Source data

Additionally, knockout of ILVBL or knockdown of ALDH3A2 increased LD abundance in HeLa cells under GS, with combined loss of both proteins resulting in even greater accumulation of LDs (Fig. 4k). To assess the roles of ILVBL and ALDH3A2 in mitochondrial lipid catabolism, we measured fatty-acid-driven respiration (oxygen consumption in glucose-free medium with oleate). Mitochondrial oxygen consumption was quantified under basal and FCCP-stimulated conditions to evaluate both basal and maximal FAO capacities. Depletion of either enzyme reduced basal, maximum and potential mitochondrial FAO, with combined loss resulting in a more pronounced defect (Fig. 4j). Furthermore, UBQLN2 overexpression elevated LD abundance under GS, and this effect was suppressed by loss of either ILVBL or ALDH3A2 and completely abolished when both were absent (Fig. 4k and Extended Data Fig. 7a). Together, these results identify ILVBL and ALDH3A2 as essential UBQLN2 effectors that sustain mitochondrial FAO and lipid homeostasis under energy stress.

### ALS/FTD-linked UBQLN2 mutations disrupt ILVBL and ALDH3A2 turnover and lipid homeostasis

The proteomic turnover analysis showed that the half-lives of ILVBL and ALDH3A2 were significantly prolonged in iMNs harboring ALS/FTD-linked UBQLN2 mutations (Fig. 4d,e), suggesting that dysregulation of the UBQLN2–ILVBL/ALDH3A2 axis is associated with these disease mutations. This aberrant protein turnover was further validated by cycloheximide chase assays in neurons (Extended Data Fig. 7b,c). Consistently, ectopic expression of UBQLN2 mutants in HeLa cells led to substantially higher ILVBL and ALDH3A2 levels than wild-type (WT) UBQLN2 (Extended Data Fig. 7d). Mechanistically, ALS/FTD-linked UBQLN2 mutations weaken the interaction between UBQLN2 and ILVBL and accelerate the degradation of mutant UBQLN2 under GS, resulting in the loss of UBQLN2 function through both impaired substrate binding and increased self-turnover (Extended Data Fig. 7b,c,e,f and Supplementary Table 1).

**Extended Data Fig. 8:**
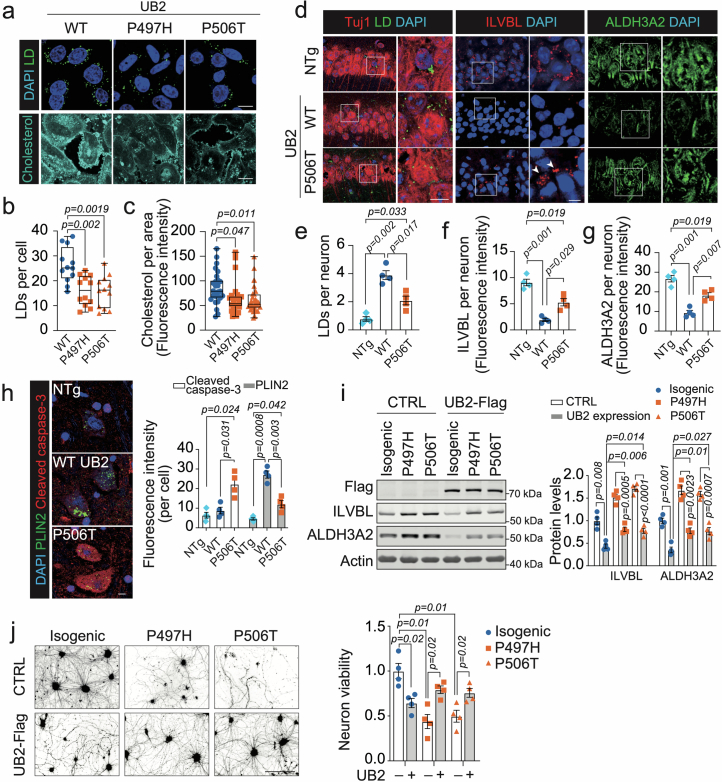
Disease-linked mutant UBQLN2 exhibits loss of function in regulating ILVBL, ALDH3A2, and lipid metabolism. **a**-**c**, BODIPY and Filipin III staining shows increased LDs and decreased cholesterol levels in HeLa cells expressing mutant UBQLN2 compared to wild-type. Cells were transfected with indicated plasmids for 24 hours and cultured in CM for another 24 hours. The number of LDs per cell (**d**) or cholesterol fluorescence intensity (**f**) per area was statistically analyzed (for **b**, n = 12 fields of 89 cells for wild-type UB2, 77 cells for P497H, 81 cells for P506T; for c, n = 27 fields of 98 cells for wild-type UB2, n = 25 fields of 89 cells for P497H, and n = 30 fields of 113 cells for P506T cells. Data were collected from three independent experiments). Scale bar, 10 μm. **d**-**g**, Representative images of LDs, ILVBL, ALDH3A2 in hippocampal neurons in transgenic mice with neuron-specific expression of human wild-type or mutant UBQLN2. Each dot represents the average number of LDs or the average fluorescence intensity of a target protein per neuron in a brain (n = four mice). Scale bar, 10 μm. **h**, Immunostaining images show reduced LDs (PLIN2) with elevated cleaved caspase-3 in the spinal cord neurons of UBQLN2P506T transgenic mice. The fluorescence intensity of PLIN2 and cleaved caspase-3 in each neuron was analyzed, and 23 to 45 neurons were analyzed in each mouse of the groups (n = four mice). Scale bar, 10 μm. **i**, Immunoblotting analysis of ILVBL and ALDH3A2 protein levels in UBQLN2 mutant iMNs and isogenic controls, with or without ectopic expression of UBQLN2. iMNs were infected with lentivirus particles to express UBQLN2 for three days (n = three biological replicates in three independent experiments). **j**, Calcein-AM staining demonstrates reduced neuronal viability of UBQLN2 mutant iMNs under GS, a deficit that was alleviated by re-expression of wild-type human UBQLN2. iMNs were infected with lentivirus for three days and then cultured in glucose-free medium for an additional three days (n = four biological replicates in two independent experiments). Scale bar, 100 μm. Group differences were assessed by the Brown-Forsythe and Welch ANOVA test with a post-hoc analysis of Dunnett’s test (two-sided) for panels **b**-**i**. For panel **j**, the group differencese were assessed by Brown-Forsythe and Welch ANOVA test with Benjamini-Krieger-Yekutieli two-stage step-up procedure (two-sided). For panels **b** and **c**, box plots are presented as minima to maxima. For panels **e**-**j**, data are presented as mean ± SEM. Source data

We next assessed how these UBQLN2 mutations affect lipid metabolism. Compared to controls, HeLa cells expressing UBQLN2 mutants led to reduced intracellular LD and cholesterol levels (Extended Data Fig. 8a–c), indicating that the disease-linked mutations compromise the ability of the cell to maintain cellular lipids. To study UBQLN2 function in vivo, we used transgenic mice expressing WT or P506T mutant UBQLN2 by the neuron-specific Thy1.2 promoter. The UBQLN2^P506T^ mice, but not the UBQLN2^WT^ transgenic mice, developed motor and memory deficits due to neuronal loss in the spinal cord and hippocampus^33^. Compared to non-transgenic mice, UBQLN2^WT^ mice had elevated levels of LDs and reduced levels of ILVBL and ALDH3A2 in hippocampal neurons (Extended Data Fig. 8d–g), confirming the role of UBQLN2 in promoting ILVBL/ALDH3A2 degradation and LD accumulation in vivo. By contrast, UBQLN2^P506T^ mice exhibited significantly lower LD abundance and elevated ILVBL and ALDH3A2 protein levels in hippocampal neurons (Extended Data Fig. 8d–g). Furthermore, immunostaining demonstrated LD deficiency, as reflected by the reduced level of the LD protein marker PLIN2 and elevated levels of cleaved caspase-3 in the spinal cords of UBQLN2^P506T^ mice compared to UBQLN2^WT^ mice (Extended Data Fig. 8h), linking LD depletion to neuronal loss in vivo.

To test whether restoring UBQLN2 function could enhance neuronal viability, we expressed similar levels of WT UBQLN2 via lentiviruses in P497H or P506T mutant iMNs and their isogenic controls (Extended Data Fig. 8i). WT UBQLN2 expression significantly improved survival in the mutant iMNs while reducing neuronal survival in the isogenic control iMNs under GS (Extended Data Fig. 8j). Accordingly, the ectopic expression of UBQLN2 alleviated the accumulation of ILVBL in the mutant iMNs but led to ILVBL depletion in the isogenic controls (Extended Data Fig. 8i). These data indicate that an optimal level of UBQLN2 activity is crucial for maintaining the stability of its client proteins and overall neuronal fitness.

### Knockdown of ILVBL or ALDH3A2 mitigates neurodegeneration in mice expressing mutant UBQLN2

**Fig. 5:**
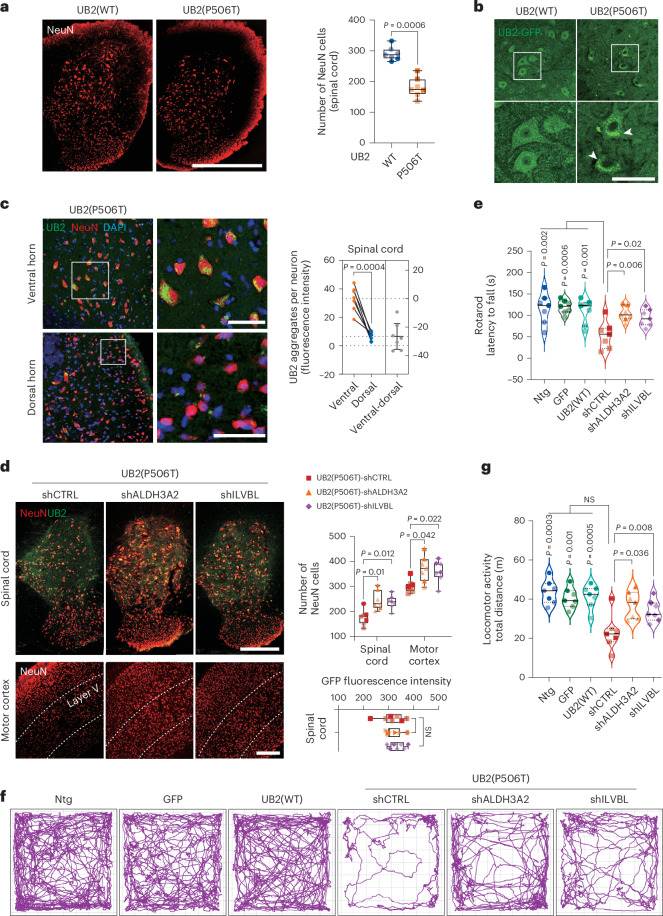
Knockdown of ILVBL or ALDH3A2 alleviates mutant UBQLN2-induced neurodegeneration and behavioral deficits in vivo*.* **a**, Representative NeuN immunostaining of spinal cord sections from mice expressing WT or P506T mutant UBQLN2 5 months after AAV-PHP.eB delivery. Quantification reveals significant neuronal loss in UBQLN2(P506T) mice (*n* = 7 mice; three males and four females). Scale bar, 500 μm. **b**,**c**, UBQLN2(P506T), but not WT UBQLN2, formed visible aggregates in spinal cord neurons (**b**), preferentially accumulated in ventral horn neurons (**c**). White arrowheads in **b** indicate perikaryal inclusions (*n* = 7 mice; three males and four females. The fluorescence intensity of UBQLN2 aggregates per neuron was analyzed; scale bar, 100 μm). Data are presented as paired differences (ventral-dorsal) with the mean effect size and 95% confidence interval. **d**, shRNA-mediated knockdown of ALDH3A2 or ILVBL in UBQLN2(P506T) mice attenuated neuronal loss in both the spinal cord and layer V of the motor cortex without reducing UBQLN2 aggregation (*n* = 7 mice; three males and four females). Scale bar, 200 μm. **e**–**g**, Rotarod tests (**e**) and open-field tracks (**f**,**g**) show impaired motor coordination and reduced spontaneous locomotion in UBQLN2(P506T) mice. These deficits were mitigated by knockdown of ILVBL or ALDH3A2 (*n* = 7 mice; three males and four females). Statistical analyses for **a** and **c** were performed using an unpaired and paired two-tailed Student’s *t*-test, respectively. For **a** and **c**, statistical differences were assessed, using an unpaired and paired Mann–Whitney *U*-test (two-sided), respectively. For **d**, **e** and **g**, statistical significance was assessed with the Kruskal–Wallis test, with multiple comparisons controlled using the Benjamini–Krieger–Yekutieli two-stage step-up procedure (two-sided). Box plots are shown in minima to maxima for **a** and **d**. NS, not significant. Source data

**Extended Data Fig. 9:**
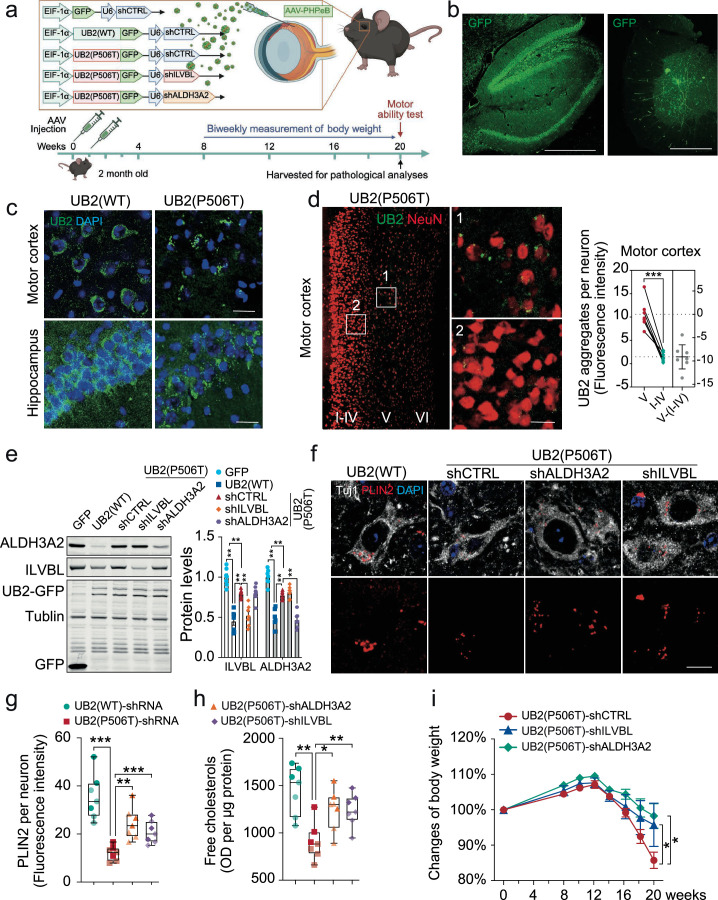
Knockdown of ILVBL or ALDH3A2 ameliorates UBQLN2(P506T)-induced lipid dysregulation in mice. **a**, Experimental timeline and AAV vectors designed for expressing UBQLN2(P506T)-GFP and simultaneous shRNA targeting of ILVBL or ALDH3A2 via AAV-PHP.eB retro-orbital injection. **b**, Representative images showing robust expression of UBQLN2-GFP in the hippocampus and spinal cord five months after AAV injection. Scale bar, 500 μm. **c** and **d**, Immunostaining demonstrates that UBQLN2(P506T), but not wild-type UBQLN2, formed aggregates in the hippocampus and motor cortex layer V (n = seven mice; three males and four females). Scale bar, 50 μm. **e**, Immunoblot validation of expression of wild-type or mutant UBQLN2-GFP and effective knockdown of ILVBL or ALDH3A2 in spinal cords (n = seven mice; three males and four females). **f**-**h**, PLIN2 immunostaining (**f**, **g**) and colorimetric quantification of free cholesterol (**h**) indicate metabolic improvement in UBQLN2(P506T) mouse spinal cords following depletion of ILVBL or ALDH3A2 (n = seven mice; three males and four females. Scale bar, 10 μm). **i**, Body weight trajectory from 8 to 20 weeks after AAV injection reveals a progressive decline in UBQLN2(P506T) mice, a deficit that was mitigated by knockdown of ILVBL or ALDH3A2 (n = seven mice; three males and four females). A paired two-tailed Student’s t-test was performed for the statistical analysis of panel **d**. Group differences in panels **e**, **g**, and **h** were assessed by the Brown-Forsythe and Welch ANOVA test with a post-hoc analysis of Dunnett’s test (two-sided). For the statistical analysis of panel **i**, two-way ANOVA followed by Tukey’s post-hoc test (two-sided) was performed to assess the effects of genotype and time. For panels **d**, **e** and **i**, data are presented as mean ± SEM. For panels **g** and **h**, box plots are presented as minima to maxima. Schematic in **a** created in BioRender; Liu, Y. https://biorender.com/7njkzj4 (2025). Source data

To evaluate the in vivo roles of ILVBL and ALDH3A2 in UBQLN2-linked neurodegeneration, we developed an adeno-associated virus (AAV)-based ALS mouse model using AAV-PHP.eB, a capsid variant optimized for central nervous system (CNS) transduction^52^, to co-express mutant UBQLN2 and shRNAs targeting ILVBL or ALDH3A2 (Extended Data Fig. 9a). Retro-orbital delivery resulted in robust CNS transduction, as verified by green fluorescent protein (GFP) expression in the brain and spinal cord (Extended Data Fig. 9b). Approximately 5 months after injection, mice expressing UBQLN2 (P506T) exhibited impaired motor performance compared to GFP or WT UBQLN2 controls (Fig. 5e–g), accompanied by neuronal loss in the spinal cord (Fig. 5a). Consistent with patient pathology^25^, UBQLN2 (P506T) formed abundant aggregates in the spinal cord, cortex and hippocampus (Fig. 5b and Extended Data Fig. 9c). Because motor neurons are selectively affected in patients with ALS, we focused on the spinal cord and cortical layers V and VI, which are regions enriched in upper motor neurons and responsible for motor learning^53,54^. TUBQLN2 (P506T) aggregates were concentrated in ventral horn neurons of the spinal cord and in cortical layers V–VI, whereas superficial cortical layers were relatively spared (Fig. 5c). Thus, this AAV model recapitulates the key behavioral and neuropathological features of UBQLN2-associated ALS/FTD.

We then tested whether reducing ILVBL or ALDH3A2 expression could mitigate the disease phenotypes in vivo. Immunoblotting confirmed greater than 50% knockdown efficiency for both ILVBL and ALDH3A2 in mouse spinal cords (Extended Data Fig. 9e). In AAV-PHP.eB P506T UBQLN2 mice, knockdown of either ILVBL or ALDH3A2 significantly increased neuronal survival and elevated LD and free cholesterol levels in the spinal cord, without altering mutant UBQLN2 aggregation (Fig. 5d and Extended Data Fig. 9f–h). A similar neuroprotective effect was observed in cortical layer V (Fig. 5d). Behaviorally, ILVBL or ALDH3A2 knockdown improved motor performance of P506T mice in rotarod and open-field assays (Fig. 5e–g) and increased their body weight, indicating a broader physiological benefit (Extended Data Fig. 9i). Together, these results support ILVBL and ALDH3A2 as key effectors mediating mutant UBQLN2-induced neurodegeneration in vivo.

### TDP-43 proteinopathy disrupts the UBQLN2−ILVBL/ALDH3A2 axis and lipid metabolism

**Extended Data Fig. 10:**
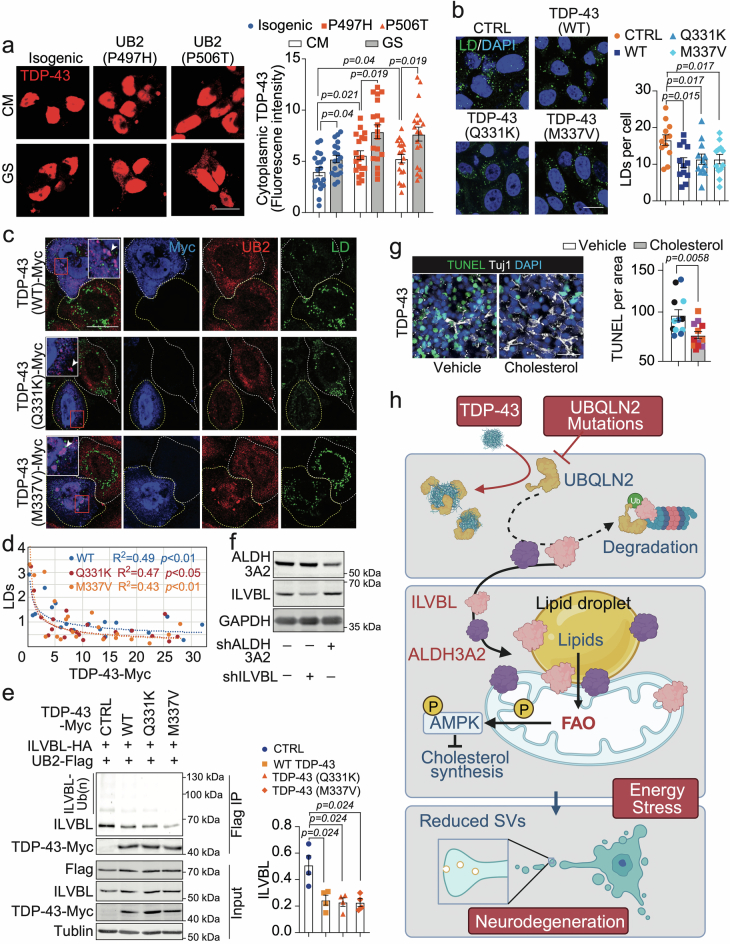
TDP-43 proteotoxicity disrupts UBQLN2-mediated lipid metabolism. **a**, Immunostaining for TDP-43 in isogenic and UBQLN2 mutant motor neurons cultured in CM or under GS for 24 hours (Scale bar, 10 μm). (n = 20 neurons per group). **b**, BODIPY staining of lipid droplets (LDs) reveals LD depletion in HeLa cells expressing TDP-43 (wild-type, Q331K or M337V) compared to the control vector under GS. HeLa cells were transfected with the indicated plasmids for 24 hours and cultured in glucose-free medium for an additional 24 hours. (n = 12 fields in three independent experiments). Each dot represents the average number of LDs per cell in a field. Scale bar, 10 μm. **c** and **d**, Immunofluorescence analyses show disrupted LD maintenance and colocalization of UBQLN2 with cytoplasmic TDP-43 under GS. Quantification indicates an inverse correlation between cytoplasmic TDP-43 signal intensity and LD number per cell (n = 11-20 cells in two independent experiments). Each dot represents the number of LDs in a single cell. **e**, Co-immunoprecipitation analyses show reduced interaction between UBQLN2 and ILVBL upon the ectopic expression of wild-type or mutant TDP-43 (Q331K, M337V). HeLa cells were transfected with UBQLN2-Flag and wild-type or mutant TDP-43-Myc (n = three biological replicates from three independent experiments). **f**, Immunoblotting confirms knockdown of ILVBL and ALDH3A2 in wild-type and TDP-43 mutant iMNs. The experiments were repeated twice with two independent shRNAs. **g**, Cholesterol supplementation reduced apoptosis in TDP-43 mutant organoids under GS. Neuronal apoptosis was measured by TUNEL staining in COs carrying the TDP-43 mutation (A382T, G298S, or M337V) and treated with cholesterol (1 μg/ml) under GS for 3 days (n = 12 replicates from two cell lines in two independent experiments). The purple, orange, and red dots indicate the A382T, G298S, and M337V mutant lines, respectively; while the black, dark blue, and light blue dots represent the corresponding control lines for these mutants. Scale bar, 20 μm. **h**. A model illustrating the role of UBQLN2 in coordinating lipid droplet–mitochondria fatty acid metabolism and its dysregulation in ALS/FTD. Under physiological conditions, UBQLN2 maintains lipid homeostasis by mediating the proteasomal degradation of the enzymes ILVBL and ALDH3A2. These enzymes break down lipids for mitochondrial fatty acid β-oxidation (FAO). Upon UBQLN2 dysfunction due to either ALS/FTD-linked UBQLN2 mutations or TDP-43 proteinopathy, the degradation of ILVBL and ALDH3A2 is impaired, leading to their accumulation at lipid droplets (LDs) and mitochondria and overactivation of mitochondrial FAO. This metabolic shift activates AMPK, suppresses cholesterol biosynthesis, and disrupts synaptic vesicle (SV) biogenesis, ultimately contributing to neurodegeneration in ALS/FTD. Group differences in panels **a**, **b**, and **e** were assessed by the Brown-Forsythe and Welch ANOVA test with Benjamini-Krieger-Yekutieli two-stage step-up procedure (two-sided). For panel **g**, group differences were assessed by an unpaired Mann-Whitney U test (two-sided). Data are presented as mean ± SEM for panels **a**, **b**, and **e**. Schematic in **h** created in BioRender; Liu, Y. https://biorender.com/e2bcdj3 (2025). Source data

WT UBQLN2 functions as a protein quality control factor that facilitates the clearance of TDP-43 and other neurodegeneration-associated proteins^25,30,51,55^. However, the impact of disease-associated proteotoxicity on the function of WT UBQLN2 remains unclear. TDP-43 proteinopathy, characterized by proteinaceous inclusions of WT TDP-43, is a common pathology in ALS, FTD and a subset of Alzheimer’s disease cases. Consistent with previous reports^25,33^, we observed cytoplasmic mislocalization of TDP-43 in UBQLN2 mutant iMNs compared to isogenic controls (Extended Data Fig. 10a). Additionally, GS stress induced cytoplasmic mislocalization of TDP-43 in both isogenic and UBQLN2 mutant iMNs (Extended Data Fig. 10a), suggesting that metabolic stress may contribute to TDP-43 proteinopathy.

**Fig. 6:**
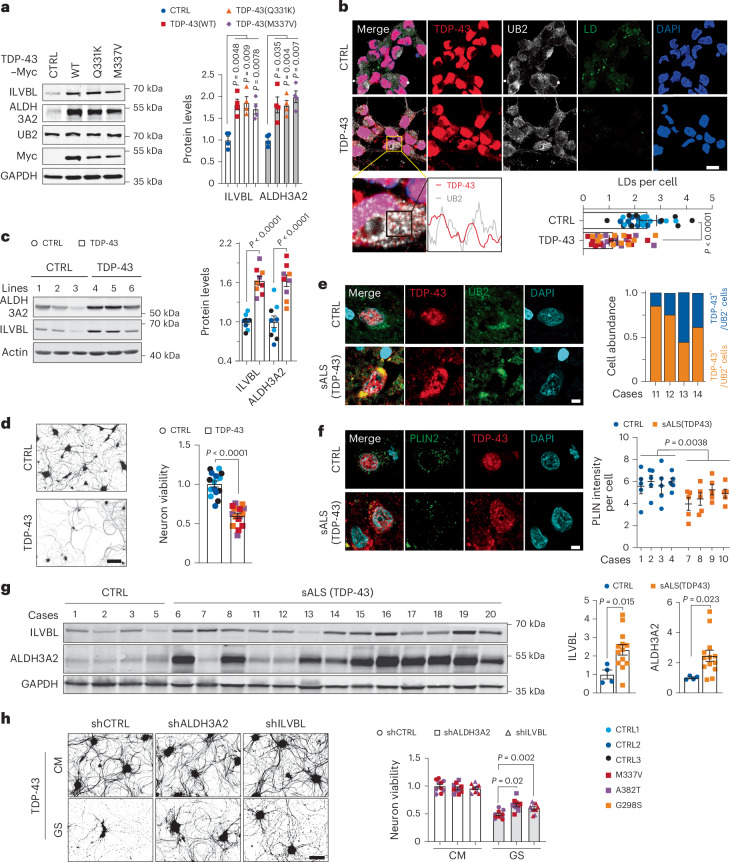
TDP-43 proteinopathy compromises UBQLN2-mediated regulation of ILVBL and ALDH3A2 and disrupts lipid metabolism. **a**, Immunoblotting analyses show upregulation of ILVBL in HeLa cells ectopically expressing WT or mutant TDP-43 (Q331K and M337V) for 24 hours, followed by an additional 24 hours of GS (*n* = 3 biological replicates in three independent experiments). **b**, Immunofluorescence staining of iMNs demonstrates co-localization of UBQLN2 with TDP-43 (WT, A382T or G298S), accompanied by LD depletion. LDs were labeled with BODIPY(493/503) (*n* = 30 fields of 91 neurons for control and *n* = 30 fields of 147 neurons from three mutant cell lines and three control lines in three independent experiments; scale bar, 10 μm). The purple, orange and red dots indicate the A382T, G298S and M337V mutant lines, respectively, and the black, dark blue and light blue dots represent their corresponding control lines, as indicated in **h**. **c**, Immunoblotting analyses show increased ALDH3A2 and ILVBL protein levels in TDP-43 mutant iMNs relative to controls across multiple cell lines (*n* = 3 biological replicates of three pairs of mutant and control lines in three independent experiments). The color-coded dots correspond to those in **b**. **d**, Calcein-AM staining reveals reduced viability of TDP-43 mutant iMNs under GS for 3 days (*n* = 5 biological replicates of three pairs of mutant and control cell lines; scale bar, 200 μm). The color-coded dots correspond to those in **b**. **e**,**f**, Spinal cord sections from patients with sALS with TDP-43 pathology showed cytoplasmic co-localization of UBQLN2 with TDP-43 aggregates (**e**) and decreased LD abundance (**f**, PLIN2 as an LD marker) in neurons (*n* = 4 patients or non-neurological controls, with 20 neurons in each group). Scale bar, 10 μm. **g**, Immunoblotting of spinal cord tissue from patients with sALS shows increased accumulation of ILVBL and ALDH3A2 proteins (*n* = 4 for control and *n* = 13 for patient cases). **h**, Silencing ILVBL or ALDH3A2 restored the viability of TDP-43 mutant iMNs under GS for 3 days, as shown by Calcein-AM staining (*n* = 5 biological replicates of two mutant cell lines; scale bar, 100 μm). For **a** and **h**, group differences were assessed by Brown–Forsythe and Welch ANOVA test with a post hoc analysis of Dunnett’s test (two-sided). **b**–**d**, **f** and **g** were analyzed using an unpaired Mann–Whitney *U*-test (two-sided). For **a**–**d** and **f**–**h**, data are presented as mean ± s.e.m. CTRL, control. Source data

We investigated how TDP-43-associated proteotoxicity affects the UBQLN2–ILVBL/ALDH3A2 axis and lipid metabolism. Under GS, overexpression of WT or ALS-linked TDP-43 variants (Q331K and M337V) increased ILVBL and ALDH3A2 protein levels (Fig. 6a) and reduced LD abundance in HeLa cells (Extended Data Fig. 10b). Because TDP-43 overexpression did not alter UBQLN2 abundance (Fig. 6a), we asked whether it impairs the function of UBQLN2 through sequestration. Indeed, ectopic expression of either WT or mutant TDP-43 led to cytoplasmic inclusions that co-localized with UBQLN2 and were accompanied by LD depletion (Extended Data Fig. 10c,d). Co-immunoprecipitation further showed that both WT and mutant TDP-43 interact with UBQLN2, thereby disrupting UBQLN2–ILVBL binding (Extended Data Fig. 10e). Together, these results suggest that TDP-43 interferes with UBQLN2’s interactions with its client proteins, compromising its function in regulating lipid metabolism.

We examined the effects of TDP-43 proteotoxicity on the UBQLN2–ILVBL/ALDH3A2 axis in patient-derived motor neurons. Although control iMNs showed minimal cytoplasmic TDP-43, iMNs from patients with ALS harboring mutant TDP-43 (A382T, G298S or M331V) exhibited prominent cytoplasmic aggregates under GS (Fig. 6b). Consistent with previous reports linking protein quality control factors to TDP-43 inclusions^56^, we observed that most of the TDP-43 aggregates co-localized with UBQLN2 (Fig. 6b), indicating recruitment of UBQLN2 to cytoplasmic TDP-43 aggregates. These mutant iMNs exhibited LD depletion and elevated ILVBL and ALDH3A2 levels compared to controls (Fig. 6b,c). Moreover, they showed significantly reduced viability under 3 days of GS, suggesting increased vulnerability to metabolic stress (Fig. 6d).

We then analyzed the pathologies related to the UBQLN2–ILVBL/ALDH3A2 axis in spinal cord tissues from patients with sporadic ALS (sALS) with TDP-43 proteinopathy. Immunostaining revealed that control tissues showed no cytoplasmic distribution of TDP-43, whereas ALS patient samples exhibited cytoplasmic TDP-43 inclusions (Fig. 6e). Notably, more than half of these inclusions were UBQLN2 positive (Fig. 6e). Additionally, LDs marked by PLIN2 were depleted, and ILVBL and ALDH3A2 levels were substantially increased in the ALS spinal cord tissues (Fig. 6f,g). These results suggest that TDP-43 proteinopathy disrupts the UBQLN2–ILVBL/ALDH3A2 axis, contributing to impaired proteostasis and lipid dysregulation in ALS.

Lastly, to test whether the dysregulation of UBQLN2 client proteins was involved in TDP-43 neurotoxicity, we knocked down ILVBL or ALDH3A2 in TDP-43 mutant iMNs (Extended Data Fig. 10f). Silencing either gene significantly enhanced neuronal survival in TDP-43 mutant iMNs subjected to GS for 3 days (Fig. 6h). Additionally, cholesterol supplementation (1 μg ml^−1^) in TDP-43 mutant COs significantly reduced neuronal apoptosis (Extended Data Fig. 10g). These results demonstrate that the heightened vulnerability of TDP-43 mutant neurons to metabolic stress can be alleviated by targeting lipid dysregulation through ILVBL, ALDH3A2 or cholesterol restoration.

## Discussion

Impaired protein homeostasis is one of the most common features in neurodegenerative diseases, and dysregulation in lipid metabolism is increasingly recognized as a key contributor to neurodegeneration^40,57,58^. Here we identify UBQLN2 as a central node connecting proteostasis and lipid metabolism in ALS/FTD (Extended Data Fig. 10h). UBQLN2 governs lipid metabolism by promoting the degradation of lipid-catabolizing enzymes ILVBL and ALDH3A2. This regulation of lipid metabolism is essential for synaptic vesicle maintenance and neuronal survival, especially under metabolic stress. Disease-linked UBQLN2 mutations and TDP-43 proteinopathy impair these functions, whereas inhibiting ILVBL or ALDH3A2, or restoring cholesterol levels, ameliorates neurodegenerative phenotypes and synaptic or viability defects in UBQLN2 mouse models, TDP-43 iMNs and motor neuron or organoid cultures, highlighting lipid dysregulation as a key pathogenic driver.

Dysregulated lipid metabolism has been implicated in neurodegenerative diseases such as ALS and FTD^57^. LDs serve as essential lipid reservoirs that support mitochondrial metabolism, particularly under stress conditions, through dynamic contacts between LDs and mitochondria^19,20,39^. ILVBL and ALDH3A2 are enzymes that sequentially catalyze fatty acids for oxidation in the endoplasmic reticulum and peroxisomes^50^. In the present study, we found that ILVBL and ALDH3A2 localize to both LDs and mitochondria, particularly under stress conditions, suggesting that they mediate lipid metabolism at the interface of these organelles. UBQLN2 regulates this process by targeting ILVBL and ALDH3A2 for degradation, thereby maintaining lipid metabolic homeostasis. ALS/FTD-linked mutations in UBQLN2 impair the degradation of these enzymes, resulting in metabolic imbalance. In addition to inducing oxidative stress and mitochondrial damage^20,59^, hyperactive FAO represses cholesterol biosynthesis through the accumulation of long-chain fatty acyl-CoA, an activator of the AMPK pathway^47,48^. Consistent with this mechanism, we observed that reduced cholesterol levels contribute to synaptic vesicle loss. These findings establish UBQLN2 as a critical regulator that integrates synaptic homeostasis, LD dynamics and mitochondrial metabolism in the context of neurodegeneration.

UBQLN2 is increasingly recognized as a key player in broader neurodegenerative pathologies, owing to its molecular chaperone functions in multiple proteinopathies, including the TDP-43 proteinopathy, in diverse neurodegenerative conditions^25,34,35^. Our findings demonstrate that TDP-43 proteotoxicity compromises the ability of UBQLN2 to regulate ILVBL and ALDH3A2 turnover, illustrating how proteinopathy can impair the metabolic functions of UBQLN2. This disruption may underlie key neurodegenerative features, such as mitochondrial dysfunction and synaptic impairment, commonly observed in proteotoxicity-associated diseases^60–62^. The realization of UBQLN2 as a coordinator of protein quality control with metabolic balance provides critical insight into the maintenance of cellular homeostasis under both physiological and pathological conditions.

## Methods

### Plasmids

The sequences for all shRNAs and CRISPR gRNAs are provided in Supplementary Information. To construct plasmids for expressing hILVBL and hALDH3A2 in mammalian cells, the coding sequences of human *ILVBL* (NM_006844) and *ALDH3A2* (BC002430) were amplified from human cDNA by polymerase chain reaction (PCR) using a Taq Master Mix kit (Vazyme, P222-01). The resulting PCR products were cloned into mammalian expression vectors: ILVBL into pCMV6-Entry with a C-terminal Myc–DDK tag and ALDH3A2 into pCMV3-SP-N-HA. WT, UBA-deleted, UBL-deleted, PXX-deleted, P497T or P506T UBQLN2 was cloned into a pcDNA3.1 vector with a 3×Flag tag. WT, Q331K or M337V TDP-43 was cloned into a PRK5 vector containing an N-terminal Myc tag (EQKLISEEDL)^63^. For neuronal expression, WT UBIQLN2 was cloned into a pLenti CMV Puro DEST (w118-1, RRID: Addgene_107502), a gift from Alejandro Gutierrez (RRID: Addgene_107507)^64^.

### Cell culture, nutrient stress and survival tests

Human iPSCs were cultured in StemFlex medium (Gibco, A3349401), with medium exchanged every other day. HeLa and HEK293 cells were maintained in DMEM containing 10% FBS. Cell lines were checked regularly for mycoplasma contamination using a PCR-based detection kit (MilliporeSigma, MP0025). For GS stress, HeLa and HEK293 cells were cultured in glucose-free DMEM (Gibco, 11966025) containing 10% dialyzed FBS (Gibco, 26400044) for the indicated time. Transient transfection using Lipofectamine 2000 was used for all gene knockdown and cDNA expression in HeLa and HEK293 cells. In brief, cells with 60% confluence were incubated with plasmids in Opti-MEM (Gibco, 51985091) for 8 hours. After transfection, cells were maintained in DMEM containing 10% FBS overnight, followed by treatments and measurements. Cell survival for HeLa cells was examined by crystal violet staining^65^. In brief, cells were fixed in methanol for 15 minutes and stained with 0.1% crystal violet for 20 minutes. The crystal violet dye was dissolved in 10% acetic acid, and the optical densities were measured at 570 nm using a Synergy H1 Hybrid Reader (BioTek). All experiments were performed at room temperature.

### Mice

The genotyping of transgenic mice expressing WT and P506T human UBQLN2 driven by a Thy1.2 promoter was described previously^33^. After being backcrossed with C57BL/6 mice (The Jackson Laboratory, stock no. 000664) for at least 10 generations, the transgenic mice were used in this study. Mice subjected to the study were all at the age of 52 weeks. All animal procedures were approved by the University of Maryland Baltimore Animal Care and Use Committee and conducted in full accordance with the National Institutes of Health (NIH) Guide for the Care and Use of Laboratory Animals.

For AAV injection experiments, 8-week-old WT C57BL/6 mice (*n* = 7; four females and three males) were used. Mice were randomly assigned to experimental groups, and all outcome assessments were performed by investigators blinded to genotype and treatment. All animal procedures were approved by the Institutional Animal Care and Use Committee of Johns Hopkins University and were conducted in accordance with the NIH Guide for the Care and Use of Laboratory Animals.

### AAV-PHP.eB preparation and in vivo treatment

AAV vectors were produced in HEK293T cells using a triple-plasmid transfection protocol followed by chloroform-based purification, as described previously^66^. In brief, a total of 1.1 × 10^7^ HEK293T cells were seeded per 175-cm^2^ flask and transfected at 75–80% confluency with equimolar amounts of pHelper (Addgene, 112867), AAV2 rep-AAV-PHP.eB cap (Addgene, 103005) and transgene plasmids using Lipofectamine 2000 (Invitrogen, 2 µg of Lipofectamine per 1 µg of DNA). Twenty-four hours after transfection, the culture medium was replaced with OptiPRO serum-free medium. After an additional 72 hours, cells and supernatants were harvested and lysed by chloroform extraction, followed by viral precipitation using PEG-8000 and resuspension in HEPES buffer. Nuclease treatment (DNase I, RNase A and MgCl_2_) was performed to remove contaminating nucleic acids. The aqueous viral phase was further purified by repeated chloroform extraction and ultrafiltration using 100-kDa Amicon Ultra centrifugal filters. The AAV was collected, resuspended in DPBS and stored at 4 °C for up to 6 months. Vector genome titers were determined by the SYBR Green-based quantitative PCR (qPCR) method^67^. In brief, AAV samples were treated with DNase I (37 °C, 30 minutes) to degrade unpackaged DNA, followed by heat inactivation. Viral capsids were then disrupted by incubation at 95 °C for 10 minutes. qPCR was performed using primers targeting the transgene cassette (for example, WPRE or CMV), with a standard curve generated from serial dilutions of a plasmid containing the same target sequence. Reactions were run using SYBR Green qPCR Master Mix, and viral genome titers were calculated based on the standard curve and expressed as genome copies per milliliter.

Eight-week-old C57BL/6 mice were anesthetized with isoflurane and administered AAV vectors via retro-orbital sinus injection using a 31-gauge insulin syringe. Each eye of mouse received 50 μl of viral suspension containing 1 × 10^12^ vector genomes per injection. One week later, this injection was repeated to enhance transduction efficiency. Animals were monitored during recovery and returned to their home cages.

### Motor behavior assays

Motor coordination and balance were assessed using an accelerating rotarod apparatus. Seven mice—three males and four females—were first habituated to the apparatus over two consecutive days, during which each mouse underwent two training sessions per day (maximum 180 seconds per session) at constant low speeds. On the testing day, mice were placed on the rotarod with an initial speed of 4 r.p.m. After 1 minute, the speed was increased to 8 r.p.m. and then to 12 r.p.m. after another minute. The time each mouse remained on the rotating rod before falling was recorded as the latency to fall and used for statistical analysis. Each mouse performed three trials with at least 10 minutes of inter-trial rest to minimize fatigue.

Spontaneous locomotor activity was measured using an open-field arena (40 × 40 cm). Mice were placed individually in the center of the arena and allowed to explore freely for 10 minutes. Prior to testing, animals were habituated to the testing room for at least 30 minutes to minimize stress-induced variability. The total distance traveled was recorded and analyzed using automated tracking software and used as the primary measure of locomotor activity.

### Motor neuron differentiation, treatments and survival tests

Isogenic and mutant iPSC lines were differentiated into motor neurons following a previously established protocol^22^. In brief, iPSCs were dissociated using dispase (1 U ml^−1^), seeded onto Matrigel-coated plates and maintained in StemFlex medium with 10 μM ROCK inhibitor. The next morning, iPSCs entered the stage of neuroepithelial progenitor cells (NEPCs) by replacing the medium with neural medium (1:1 DMEM/F12:neurobasal medium, 1× GlutaMAX, 0.5× N2 supplement, 0.5× B27 supplement and 0.1 mM ascorbic acid) containing 3 μM CHIR99021, 2 μM SB431542 and 2 μM DMH-1. After differentiating for 6 days, NEPCs were dissociated with dispase (1 U ml^−1^) and split into new plates coated with Matrigel with a ratio of approximately 1:8. NEPCs were then ready to be differentiated into motor neuron progenitor cells (MNPCs) by being maintained in neural medium supplemented with 2 μM SB431542, 1 μM CHIR99021, 2 μM DMH-1, 0.1 μM retinoic acid and 0.5 μM purmorphamine for another 6 days. Then, MNPCs were dissociated and cultured in suspension using the neural medium containing 0.5 μM retinoic acid and 0.1 μM purmorphamine. Eight days later, neurospheres were dissociated into single cells and seeded onto a Matrigel-coated plate at a concentration of 2 × 10^6^ per well of a six-well plate. Adherent neurons were cultured in the motor neuron maturation medium (neural medium supplemented with 0.5 μM retinoic acid, 0.1 μM purmorphamine and 0.1 μM Compound E). After 8 days, mature iMNs were collected for experiments. The medium was changed every 2 days over the entire differentiation period. For nutrient stress of GS on iMNs, glucose-free Neurobasal medium (Gibco, A2477501) supplemented with insulin-minus B27 (Gibco, A18956) was used. For lipid treatment, mature motor neurons were cultured in glucose-free medium supplemented with cholesterol (MilliporeSigma, O1257) at indicated concentrations. On day 3 after the treatment, neuronal survival was measured by Calcein-AM staining.

Lentivirus was used to express UBQLN2 in motor neurons. To prepare the viruses, HEK293 cells were co-transfected with the lentiviral plasmid and viral packaging vectors (psPAX2 and pMD2G). Sixty hours after transfection, cell supernatants were collected and filtered through 0.45-μm cellulose acetate strainers. Lentivirus particles were concentrated in PEG8000 buffer and dissolved in PBS. Motor neurons were transduced with the lentivirus particles twice. Three days after the infection, neurons were incubated in glucose-free medium for another 3 days, and neuronal viability was measured on day 3 of the starvation by Calcein-AM staining.

### CO differentiation and treatments

COs were differentiated from human iPSCs, as previously described^68^. In brief, iPSCs were cultured in Essential 8 medium to reach approximately 70% confluency, and cells were treated with 1% DMSO for 24 hours. The next day, which is defined as day 0, cells were dissociated into single cells by incubating in Accutase (STEMCELL Technologies) after washing with PBS once. Cells were pelleted by centrifugation and resuspended in the Essential 8 medium containing 10 μM ROCK inhibitor (Y-27632). Each well in an AggreWell 800 plate, which contains 300 microwells per well, was seeded with 3 × 10^6^ cells. The plate was centrifuged at 100*g* for 3 minutes to distribute the cells at approximately 10,000 cells per microwell. Cells were cultured at 37 °C in a 5% CO_2_ incubator for 24 hours to form spheroids. After being filtered by a 40-μm cell strainer (Thermo Fisher Scientific), the retained spheroids were maintained in Essential 6 medium (Thermo Fisher Scientific, A1516401) containing dual SMAD pathway inhibitors (100 nM LDN193189 (STEMCELL Technologies, 72147) and 10 μM SB431542) and 2.5 μM XAV939 (Tocris, 3748). The medium was changed every day for 5 days. On day 6, the medium was replaced with neuronal differentiation medium consisting of Neurobasal A (Thermo Fisher Scientific, 10888-022) plus 1× B27 supplement minus vitamin A (Thermo Fisher Scientific) and 1× GlutaMAX in the presence of 20 ng ml^−1^ EGF (R&D Systems, 236-EG) plus 20 ng ml^−1^ FGF2 (R&D Systems, 233-FB). The medium was changed daily for 10 days and then every other day for an additional 9 days. On day 25, the medium was changed to a neuronal differentiation medium supplemented with 20 ng ml^−1^ NT3 (PeproTech, 450-02) and 20 ng ml^−1^ BDNF (PeproTech, 450-03), and the medium was changed every other day. From day 43, the organoids were maintained in a neuronal differentiation medium without growth factors for 2 months. Plates containing the organoids were placed on an orbital shaker at 40 r.p.m. to minimize organoid fusion from day 6. Both the isogenic and mutant organoids exhibited similar growth rates. Organoids in each group were picked out randomly for immunocharacterization at different differentiation stages. Mature organoids were starved with neuronal differentiation medium in which the neurobasal medium was replaced with glucose-free medium. To test the neuroprotection of cholesterol, glucose-starved organoids were fed with cholesterol at a concentration of 1 μg ml^−1^. After being starved for 3 days, organoids were sliced for staining for LDs, apoptotic neurons and synaptic vesicles.

### Lipidomic profiling

Cell samples were homogenized, and the protein levels were quantified with the Bicinchoninic Acid (BCA) Protein Assay Kit (Pierce, 23225). Protein-normalized cell homogenates were extracted following a modified Bligh and Dyer procedure to harvest a crude lipid fraction^69^. In brief, 20 μl of cell homogenates was gently mixed in a glass vial with 980 μl of ddH_2_O and 2.9 ml of methanol:dichloromethane (2:0.9, v/v) containing the following 12 internal standards: Cer d18:1/12:0 - 6 ng ml^−1^, SM d18:1/12:0 - 0.3 ng ml^−1^, GlcCer d18:1/12:0 - 3.3 ng ml^−1^, LacCer 18:1/12:0 - 10.6 ng ml^−1^, d5-DAG d16:0/16:0 - 12.5 ng ml^−1^, d5-TAG 16:0/18:0/16:0 - 0.5 ng ml^−1^, cholesteryl-d7 ester 16:0 - 30 ng ml^−1^, PA d12:0/12:0 - 1,025 ng ml^−1^, PC 12:0/12:0 - 0.2 ng ml^−1^, PE d12:0/12:0 - 1.6 ng ml^−1^, PG d12:0/12:0 - 200 ng ml^−1^ and PS d12:0/12:0 - 900 ng ml^−1^. To obtain a biphasic mixture, an additional 1 ml of ddH_2_O and 900 μl of dichloromethane were added and vortexed. The resultant mixture was incubated on ice for 30 minutes and centrifuged (10 minutes, 3,000*g*, 4 °C) to separate the organic and aqueous phases. The organic phase was collected and stored at −20 °C. Before analysis, 600 μl of the organic layer was dried in a nitrogen evaporator (Organomation Associates) and resuspended in 150 µl of running solvent (dichloromethane:methanol (1:1) containing 5 mM ammonium acetate), and 5 mg ml^−1^ ceramide C17:0 was used to track instrument performance^70^. Lipid analysis was conducted in the MS/MSALL mode on a TripleTOF 5600 (AB SCIEX) time-of-flight (TOF) mass spectrometer. Samples (50-μl injection volume) were directly infused by high-performance liquid chromatography at a constant flow rate of 7 µl min^−1^ using an LC-20AD pump and a SIL-20AC XR autosampler (Shimazu). The mass spectrometer was operated at a mass resolution of 30,000 for TOF mass spectrometry scan and 15,000 for product ion scan in the high-sensitivity mode and automatically calibrated every 10-sample injections using APCI positive calibration solution delivered via a calibration delivery system (AB SCIEX). Source parameters were optimized and set as follows: ion source gases at 15 psi (GS1) and 20 psi (GS2), curtain gas at 30 psi, temperature at 150 °C, positive ion spray voltage at 5,500 V, declustering potential at 80 V and precursor ion collision energy at 10 V. Each sample was run in duplicate in positive ion mode. An initial TOF mass spectrometry scan provided an overview of the total lipid content at an accumulation time of 5 seconds. Precursor ions were selected by sequential 1-Thomson mass steps from 200 *m*/*z* to 1,200 *m*/*z*; the analytes in each 1-Thomson step were introduced into the collision chamber; and fragments were produced by collision-induced dissociation and identified by TOF with a scan range of 100–1,200 *m*/*z* (accumulation time of 450 ms). The collision energy for each tandem mass spectrometry (MS/MS) step was 40 V. The TOF MS and MS/MSALL data were post-aligned to internal standards using Analyst TF 1.8 (AB SCIEX) with a mass error of less than 5 ppm. The LipidView (version 1.3, AB SCIEX) database was used for the identification and annotation of lipid species based on the precursor and fragment matchings from the experimental pooled sample runs to generate a targeted lipid list to further use for lipid identification from individual experimental samples. The targeted lipid list was validated using pooled sample runs that were extracted from pooled cell homogenates and sequentially analyzed eight times. To include a lipid in the targeted list, the lipid species must be detected in seven out of eight analytical runs, and its response relative standard deviation should be below 15%. The final targeted lipid list was then used to identify the lipid species from each individual experimental sample using the targeted lipid processing MultiQuant software (version 3.0, AB SCIEX). Identified lipid intensities were normalized by their corresponding internal standard. Each sample was run in duplicate, and averaged normalized intensities for each lipid were used for statistical and bioinformatic analysis. Lipidomic molecular annotations of lipid class were integrated into our lipidomic dataset using the lipidR R package^71^. Differential lipid expression analysis was conducted using the limma R package with one-way ANOVA^72^, which applies an empirical Bayes approach to improve variance estimation. Benjamini–Hochberg correction was performed for multiple hypothesis testing across all lipids. For the analysis of each lipid category, group differences were analyzed by GraphPad Prism 10 using the Kruskal–Wallis test due to non-normal data distribution and small sample size. When significant, Dunn’s multiple comparisons test was used to compare each treatment group to the control. Significantly altered lipid species were defined as those with an adjusted *P* value (*P*_adj_) < 0.05.

### Dynamic SILAC proteomics in iMNs

After neuronal maturation, iMNs cultured in light amino acid-containing medium were gently washed with PBS twice and switched to glucose-free motor neuron maturation medium containing heavy-stable-isotope-labeled (^13^C_6_^15^N_2_) lysine (Cambridge Isotope Laboratories, CNLM-291-H-PK) and (^13^C_6_^15^N_4_) arginine (Cambridge Isotope Laboratories, CNLM-539-H-PK). Twenty-four hours later, the medium was refreshed, and iMNs were harvested exactly another 24 hours afterward (accuracy within 5 minutes). Neurons were gently washed with PBS twice, lysed in 100 µl of ice-cold lysis buffer containing 8 M urea, 50 nM Tris, 150 mM NaCl, 0.1 mM PMSF and protease inhibitor (Sigma-Aldrich, p. 8340 (1:200)), sonicated for 15 minutes and concentrated by centrifugation. Total protein concentrations were determined by NanoDrop. Protein disulfide bonds were reduced using 5 mM TCEP for 40 minutes and alkylated with 15 mM iodoacetamide for 30 minutes at 37 °C. Proteins were digested with Trypsin-LysC (Promega, V5073) at a 1:30 ratio (enzyme:protein, w/w) for 18 hours at 37 °C and quenched with 10% trifluoroacetic acid until pH < 3. Peptides were desalted using a Waters Oasis HLB 96-well extraction plate. Eluted samples were dried under SpeedVac and stored at −30 °C until liquid chromatography–mass spectrometry (LC–MS) analysis.

LC–MS analyses were conducted on a Dionex UltiMate 3000 nanoLC system coupled with a Thermo Fisher Scientific Q-Exactive HFX mass spectrometer. Samples were loaded onto an Acclaim PepMAP C18 trap column (3 µm, 100 Å, 75 µm × 2 cm) and separated on an Easy-spray PepMap C18 column (2 µm, 100 Å, 75 µm × 75 cm) with a 5-hour gradient, column temperature of 55 °C and flow rate of 0.2 µl min^−1^. The mobile phase buffer A was 0.1% formic acid in water, and buffer B was 0.1% formic acid in acetonitrile. Data-independent acquisition (DIA) LC–MS/MS analysis was conducted. Precursor scans (*m*/*z* 400–1,000) were obtained with a resolving power of 60 K, AGC of 1 × 10^6^ and MaxIT of 60 ms. Precursors were fragmented with a normalized collision energy of 30%. Peptide fragments were obtained using 75 sequential 8.0-Da staggered isolation windows with a resolving power of 15 K, MaxIT of 40 ms and 75 loop count.

Dynamic SILAC DIA proteomics data were analyzed using Spectronaut (version 17.4) software. Samples were analyzed by querying against the Swiss-Prot *Homo sapiens* database and a custom neuron-specific contaminant FASTA library^73^. Trypsin was selected as the enzyme with a maximum of two missed cleavages. A fixed modification of cysteine carbamidomethyl and a maximum of three variable modifications (methionine oxidation, acetylation of the N terminus) were allowed. Peptides between seven and 52 amino acids were included in the database search. Identifications were made with a false discovery rate (FDR) of 1%, and only y-type product ions were included in the search. All reports were filtered to remove contaminants and raw intensities below 1,000. Heavy-to-light ratios were calculated, and then the dataset was filtered to remove ratios above 100 and below 0.02. Protein half-lives were calculated using a single timepoint equation: *t*_1/2_ = *t*_s_ × (ln_2_ / ln(1 + *Ψ*)), where *t*_s_ represents the sampling time after media switch (2 days), and *Ψ* represents the heavy-to-light abundance ratio of the peptide^74^. Protein half-lives were determined using the harmonic mean of all unique peptides, as described previously^75^. A one-way ANOVA was applied to each protein to identify differences in half-lives among the three groups (isogenic control, UBQLN2-P497H and UBQLN2-P506T). To account for multiple hypothesis testing across all proteins, Benjamini–Hochberg correction was applied to the resulting *P* values, and *P*_adj_ < 0.05 was used as the threshold for significance.

### GSEA and multi-omics integration analysis

GSEA was performed using the fast GSEA (FGSEA) algorithm implemented in the fgsea R package, based on statistic outputs from differential expression analyses. Gene set annotations were obtained from multiple curated databases, including the Hallmark gene sets, Kyoto Encyclopedia of Genes and Genomes (KEGG), Reactome and Gene Ontology categories Biological Process, Cellular Component and Molecular Function. Enrichment was evaluated by the normalized enrichment score (NES), with NES > 0 indicating positive enrichment and NES < 0 indicating negative enrichment.

To investigate cross-correlations among transcriptomic (RNA sequencing (RNA-seq)), proteomic (protein half-life) and lipidomic profiles, we performed integrative analysis using the DIABLO framework within the mixOmics R package^76^. Datasets were preprocessed by removing duplicated variables and features with missing values in any samples. To comply with the <10,000 predictor variables limit required by mixOmics, RNA-seq datasets were first normalized and corrected for batch effects using DESeq2 and limma, followed by feature selection based on the 75th percentile of median absolute deviation (MAD). Low-variance features were excluded from both lipidomic and proteomic datasets. Proteins were further prioritized based on (1) a UBQLN2 interaction value that the ratio of heavy (UBQLN2) to light (control) is greater than 10 and (2) a statistically significant difference (*P*_adj_ < 0.05) between P497H and/or P506T UBQLN2 mutant compared to control.

For pathway enrichment analysis across multi-omics datasets, we applied the multiGSEA R package. Transcriptomic and proteomic features were mapped to gene symbols, and lipidomic features were annotated using Human Metabolome Database (HMDB) IDs. Enrichment analysis was then conducted using curated pathway databases, including KEGG and Reactome. Biological processes and pathways with *P*_adj_ < 0.05 were identified as significantly changed.

### Statistical analysis

Most cellular experiments were performed in triplicate across 2–3 independent biological replicates. Statistical analyses were conducted using GraphPad Prism (version 10.0) and R (version 4.4.0). For multidimensional datasets (for example, lipidomics, proteomics and transcriptomics), differential analyses were performed using the limma package^72^, Spectronaut or DESeq2 (ref. ^77^), as appropriate. Multiple hypothesis testing corrections were applied using the Benjamini–Hochberg FDR procedure, with *P*_adj_ < 0.05 considered statistically significant.

For comparisons between two groups, unpaired Mann–Whitney *U*-tests or paired two-tailed Student’s *t*-tests were used. For analyses involving more than two groups, the Kruskal–Wallis test, ordinary one-way ANOVA or Brown–Forsythe and Welch ANOVA test were applied with a Benjamini–Krieger–Yekutieli two-stage step-up procedure, Dunnett’s multiple comparisons or Dunn’s post hoc test. Two-way ANOVA followed by Tukey’s multiple comparisons test was employed to assess the interaction effects of genotype and time or dose. Data distribution was assumed to be normal, but this was not formally tested. All data were collected randomly and appropriately blocked. No statistical methods were used to predetermine sample sizes, but our sample sizes are similar to those reported in previous publications^78^. Mice were randomly divided into groups receiving different treatments. No animals or data were excluded from the analyses.

Quantification of fluorescence images was performed using ImageJ. Fluorescence intensity or puncta counts were normalized to cell number, with each field represented as a single data point in graphs. Data are presented as mean ± s.e.m. or box plots shown as minima to maxima.

The methods for generating and characterizing UBQLN2 iPSCs, lipid assays, RNA-seq, cellular staining and immunoblotting assays are provided in Supplementary Information.

### Reporting summary

Further information on research design is available in the Nature Portfolio Reporting Summary linked to this article.

## Online content

Any methods, additional references, Nature Portfolio reporting summaries, source data, extended data, supplementary information, acknowledgements, peer review information; details of author contributions and competing interests; and statements of data and code availability are available at 10.1038/s41593-026-02226-y.

## Supplementary information

Supplementary Information Parts of the methods and Supplementary Fig. 1.

Reporting Summary

Supplementary Table 1 The SILAC proteomic analysis of global protein half-lives in motor neurons derived from isogenic or UBQLN2 mutant human iPSCs.

Supplementary Table 2 Significantly enriched signal pathways in mutant iMNs under CM and GS revealed by GSEA of proteomic half-lives.

Supplementary Table 3 The lipidomic analysis of motor neurons derived from isogenic or UBQLN2 mutant human iPSCs under GS.

Supplementary Table 4 Differentially regulated genes in motor neurons derived from UBQLN2 mutant human iPSCs under GS.

Supplementary Table 5 Lipidomic analysis of HeLa cells with depletion of UBQLN2 under GS.

Supplementary Table 6 Lipidomic analysis of HEK293 cells with depletion of UBQLN2 under GS.

Supplementary Table 7 Proteins interacting with UBQLN2 identified by SILAC mass spectrometry analysis^30^.

Supplementary Table 8 ALS/FTD patients’ tissues and iPSC lines.

Supplementary Table 9 Primers for qPCRs.

## Source data

Source Data Statistical source data for all columns and graphs.

Source Data Unprocessed western blots for Figs. 4 and 6 and Extended Data Figs. 4–10.

## Data Availability

The omics data generated in this study are included in the supplementary materials (Supplementary Tables 1–6). LC–MS/MS data are deposited in the ProteomeXchange with a unique identifier: PXD048152. The raw RNA-seq data are deposited to the Gene Expression Omnibus database (GSE272994). Source data are provided with this paper.

## Code availability

No custom software codes were used to analyze the data.
