# Supplementary material for: UBQLN2 links proteotoxicity with lipid metabolism in neurodegeneration
Source: Nat Neurosci. 2026 Mar 30;29(4):782–95. doi: 10.1038/s41593-026-02226-y (PMC13058728; doi:10.1038/s41593-026-02226-y)
Supplement: Supplementary file 1 — Parts of the methods and Supplementary Fig. 1. [file 41593_2026_2226_MOESM1_ESM.pdf]

nature neuroscience

Supplementary information https://doi.org/10.1038/s41593-026-02226-y

UBQLN2 links proteotoxicity with lipid metabolism in neurodegeneration

In the format provided by the authors and unedited

Supplementary information

Methods

shRNAs and CRISPR gRNAs

shRNAs used in this study were as follows: hUBQLN2: TGTATATGACCTTAATCTTTGTGCAGCCT (shRNA-1), AACCACGAGTCCTACATCAGAATCTGGAC (shRNA-2)[1]; hALDH3a2: CCTCTGGCTCTTTATGTATTT (shRNA-1), GCATAACCATAAGCTCATCAA (shRNA-2); hILVBL: AGTCTCATCATTGCTTGCCCT (shRNA-1), CCTCTTCACGGACCCAACTGT (shRNA-2); mALDH3A2: CGTCACTTTAAGAGGTTACAA; mILVBL: GACTTTGTCTACCTGGAGTTT; and scramble shRNA: GCACTACCAGAGCTAACTCAGATAGTACT (non-targeting for human), GTTCAGATGTGCGGCGAGT (non-targeting for mouse). All DNA oligonucleotide sequences were generated by IDT. The CRISPR-Cas9 system was employed to delete ILVBL from HeLa cells using the gRNAs (TGTGCGGTTCATCTTCACGC, TGTGGTGGACACACGCCATG) that target exon 3 of ILVBL.

Generation of human iPSCs carrying ALS/FTD-linked UBQLN2 mutations

The male iPSCs carrying X-linked UBQLN2 mutations of P497H or P506T were generated in collaboration with Synthego. A small guide (sg) RNA (TGTGCTGGTGCGTGTGCTGG) was designed that contained < 2 base pair matches to other sequences in the genome to ensure precise gene targeting. The sgRNA (AGGGCCTATGGGGCCTATGG for P497H or ATGGGGGTAAAAGGGACTAT for P506T) was complexed with Cas9 protein to generate a ribonucleoprotein complex, which was nucleofected into the human PGP1 iPSC line together with single-stranded oligo DNA donors (P506T: TAGGCCCTGTAGGCCCAGTCACCCCCATAGGCCCCATAGGCCCTATAGTCACTTTTACCCCCATAGGCCCCATTGGGCCCATAGGACCCACTGGCCCTGCAGC; P497H:

TGGGGGTGGGGGTGCTGGGAACCGCTATAGGCCCTGTAGGCCCAGTCACCCACATAGGC CCCATAGGCCCTATAGTCCCTTTTACCCCCATAGGCCCCATTGG). iPSCs were dissociated and seeded at 0.5 cells/well into a 96-well dish and allowed to expand to confluency. Genomic DNAs from single iPSC clones were extracted for PCR amplification of UBQLN2 using the following primers: Forward, 5'- GCCCGCTGTTTACTGCAAAT-3'; Reverse, CCATTGCGTTGAGCTGTTCC, and screened using Sanger sequencing to confirm successful insertion.

Whole genome sequencing

The whole genome DNA of iPSCs was isolated by a genomic DNA kit (Invitrogen, K1820-01). Briefly, 8 × 106 cells were suspended in 200 µL PBS containing 20 µL Proteinase K and 20 µL RNase A followed by incubation at room temperature for two minutes. 200 µL PureLink Genomic Lysis/Binding Buffer was thoroughly mixed into the cell suspension. After incubation at 55°C for 10 minutes, 200 µL ethanol was added to the mixture and mixed well to obtain a homogeneous solution. The yield solution was loaded to a PureLink Spin Column and centrifuged at 10,000 × g for 1 minute at room temperature. After sequential washes with buffer 1 and buffer 2, the genomic DNA bound on the column was eluted in DNase-free water.

The genomic DNAs were subjected to whole genome sequencing by Novogene. Briefly, genomic DNA was randomly sheared into short fragments. The obtained fragments were end-repaired, A-tailed, and further ligated with an Illumina adapter. The fragments with adapters were PCR amplified, size selected, and purified. The library was checked with Qubit and real-time PCR for quantification. The size distribution of the library was detected by a bioanalyzer. Quantified libraries were pooled and sequenced on Illumina platforms according to effective library concentration and data amount required. The raw data were filtered to remove adaptors, low-quality nucleotides, and unrecognizable nucleotides. The cleaned reads were further

examined for sequencing error rate, GC content distribution, and sequencing quality distribution. The qualified reads were aligned to the human reference genome (hg38, http://hgdownload.cse.ucsc.edu/goldenPath/hg38/bigZips/analysisSet/hg38.analysisSet.2bit) using Burrows-Wheeler Aligner (BWA). The global profiles of genetic variants of the whole genome of each sample were visualized by Circos.

Cellular growth and pluripotency of human iPSCs

Approximately 2,500 cells per well were seeded in a 96-well plate and cultured at 37°C, 5% CO_2. Cell growth was measured every day for four days. The growth rate of human iPSCs was measured with a Cell Counting Kit-8 (Sigma-Aldrich, 96992) according to its manual. Briefly, 10 μL of the CCK-8 solution was added to each well and incubated in a cell culture incubator for 3 hours. The absorbance at 450 nm was detected using a microplate reader. The cell growth rates on different days were calculated as a ratio to the value on the first day for each cell line.

Cellular markers of stem cells and embryoid body formation were used to measure the cell pluripotency of genetically edited human iPSCs. Briefly, iPSCs were disassociated into single cells in PBS containing 0.5 mM EDTA. After centrifugation, cells were gently resuspended in an Essential 8 medium (ThermoFisher, A1517001) containing 10 μM Rock inhibitor Y27632 (Reprocell, 04-0012). 4×10^6 single cells were seeded in each well of an AggreWell 800 plate (STEMCELL Technologies, 34815). After incubation at 37 °C in a 5% CO_2 incubator overnight, the embryonic bodies formed. To measure the markers of stem cells and pluripotency, the mRNA expression of stem cell markers was measured by quantitative PCR, and primers were provided in Supplementary Table 8. Total RNAs were extracted using an RNeasy Plus mini kit (Qiagen, 74136), and cDNAs were synthesized with QuantiTect reverse transcription reagents (Qiagen, 205313). qPCR reactions were performed on a Bio-Rad thermal cycler using PowerUp SYBR Green Master Mix (ThermoFisher Scientific). The primers for pluripotent cell markers

were described previously[2,3]. The mRNA expression levels were analyzed using the ΔΔCt method and normalized against housekeeping genes.

RNA-seq analysis

Total RNAs were isolated using an RNeasy Plus mini kit (Qiagen, 74136), and RNA libraries for sequencing were constructed with a commercial kit (Invitrogen, A39005096). Briefly, RNAs were hybridized with the probe mixture at 75ºC for 5 minutes and 60ºC for 30 minutes, and rRNA was removed by incubating with rRNA depletion beads. After purification, RNAs were fragmented by RNase III to reach an average size of ~300 bp and purified by Dynabeads Cleanup Beads. After adaptor ligation, samples were reversely transcribed to synthesize a cDNA library. cDNA samples were purified and amplified by PCR. The PCR products were purified and sequenced. Raw reads were processed with TrimGalore v0.6.7[4] and controlled for quality with FastQC v.0.11.9. The processed reads were mapped to the human genome (hg38) and GENCODE v19 annotated transcripts using Hisat2 v2.2.1[5] and quantified with featureCounts v2.0.1[6]. Raw gene counts were loaded into R to perform differential expression analysis with DESeq2[7].

Lipid droplets and lipid breakdown assay

For visualizing lipid droplets, fixed cells or slides were incubated with BODIPY 493/503 dye (Invitrogen) at a dilution of 1:1,000 for 30 minutes at room temperature. After being washed in PBS three times, cells were sealed for image capture and analysis. The number of LDs per cell was quantified by ImageJ.

Lipid breakdown was measured using ^{14}C-palmitic acids (PerkinElmer, NEC534050UC) and ^{14}C-oleic acids (PerkinElmer, NEC317050UC). Specifically, HeLa cells were cultured in glucose-free medium with 0.5 μCi [^{14}C]-palmitic or [^{14}C]-oleic acids for 24 hours. Lipids were extracted by using organic extraction and centrifuge phase separation followed by drying and resuspension

(Cell Biolabs STA-612-T). The retained signals of [^{14}C] were quantified using Beckman LS6500 scintillation counter. Radioactivity was normalized by protein input levels.

Cholesterol assays

Free cholesterols were measured by Filipin III staining and a colorimetric assay dependent on the amount of red quinone, an oxidative product from free cholesterol. Filipin III staining in HeLa cells was applied using a commercial kit (Abcam, ab133116). Briefly, cells were fixed with the fixative solution for 10 minutes at room temperature, followed by three times of washes with the cholesterol detection wash buffer. Cells were incubated with filipin III at a dilution of 1:100 in cholesterol detection assay buffer for 1 hour at room temperature. After being washed twice, the fluorescence signals of cholesterol were captured by the SP8 confocal microscope (Leica) with excitation of 340-380 nm and emission of 385-470 nm. The fluorescence intensity of cholesterol per area was quantified and analyzed. For colorimetric assay of free cholesterol, HeLa cells were seeded in 6-well plates and transfected with shRNAs for 24 hours, followed by glucose starvation for an additional 24 hours. Approximately 5×10^6 cells per condition were harvested, washed with PBS, and lysed in 1 mL anhydrous ethanol by ultrasonic disruption on ice (20% power, 3 s on / 7 s off, 30 cycles). Lysates were centrifuged at 8,000 × g for 10 minutes at 4 °C, and the supernatants were collected for cholesterol quantification using a Free Cholesterol Colorimetric Assay Kit (Biorbyt, orb1173224). The assay was performed according to the manufacturer's instructions. Briefly, working reagent was prepared by mixing Reagent I and Reagent II, and then combining the resulting solution with Reagent III at a 1:3 ratio. In a 96-well plate, 5 μL of either sample, ethanol blank, or cholesterol standard (5 μmol/mL) was added to 200 μL of working reagent per well. The plate was incubated at 37 °C for 30 minutes, and absorbance was measured at 505 nm using a microplate reader. Free cholesterol concentration was calculated using the following formula: FC (μmol/10^4 cells) $=0.01 \times (\Delta A\ test / \Delta A\ standard)$,

where ΔA=A_sample – A_blank. Each condition was measured in six replicates and normalized to total protein.

For colorimetric assay of total cholesterol, HeLa cells were transfected with shRNAs for 24 hours and then cultured in glucose-free medium for an additional 24 hours. After treatment, cells (~5 × 10^6 per sample) were harvested, washed with cold PBS, and lysed in 1 mL of the provided Assay Buffer by brief sonication. Lysates were centrifuged at 12,000 × g for 10 minutes at 4 °C, and the supernatants were used for cholesterol quantification using the Total Cholesterol & Cholesteryl Ester Colorimetric Assay Kit (Abcam, ab282928) according to the manufacturer's instructions. Briefly, samples were incubated with cholesterol esterase, enzyme mix, OxiRed probe, and assay buffer at 37 °C for 60 minutes. For free cholesterol detection, the cholesterol esterase was omitted. Absorbance was measured at 570 nm using a microplate reader. Cholesteryl ester content was calculated by subtracting free cholesterol values from total cholesterol values. All measurements were performed in duplicate and normalized to total protein content as indicated.

Cholesterol uptake was quantified using the Cholesterol Uptake Assay Kit (Abcam, ab236212) according to the manufacturer's protocol. Briefly, 1 × 10^4 HeLa cells were seeded in 96-well black-walled, clear-bottom plates and allowed to adhere overnight. The next day, cells were washed twice with 1 × PBS and cultured in glucose-free medium for 24 hours. On day 3, cells were incubated with fluorescently labeled cholesterol analog (NBD-cholesterol) in serum-free medium for 1–2 hours at 37°C. After incubation, cells were washed to remove excess cholesterol, and intracellular fluorescence was measured using a microplate reader (Ex/Em = 485/535 nm). In parallel, the background signal was assessed using wells treated with uptake buffer only. Fluorescence intensity was normalized to cell number or protein content.

Immunoprecipitation, protein ubiquitination, and CHX chase assays

To measure protein degradation rates, HeLa cells were treated with cycloheximide (CHX) at 10 μg/ml under GS for 6 hours, and protein levels were measured by immunoblotting. To examine ubiquitination of UBQLN2 client proteins, HeLa cells were transfected with the protein and wild-type or mutant UBQLN2-Flag for 24 hours, followed by treatment with MG132 at 10 μM for 8 hours under GS. Cells were harvested and subjected to a pull-down assay using an antibody against Myc (Abcam, ab32, RRID: AB_303599), and the levels of ubiquitinated proteins in the precipitates were measured. To explore the role of UBQLN2 in the ubiquitination of its client protein, cells with UBQLN2 expression and MG132 treatment were subjected to two rounds of immunoprecipitation assays, with anti-Flag and anti-Myc antibodies to pull down UBQLN2 and its client protein sequentially.

For immunoprecipitation, cells were lysed in IP lysis buffer (20 mM Tris-HCl pH 7.5, 150 mM NaCl, 1 mM Na2EDTA, 1 mM EGTA, 1% Triton, 2.5 mM sodium pyrophosphate, 1 mM β-glycerophosphate, 1 mM Na_3VO_4, 1 μg/ml leupeptin, cocktail, 1 mM PMSF), sonicated for 15 minutes, and centrifuged at 14,000 × g for 10 minutes at 4 °C. The supernatants were collected for protein concentration quantification and then immunoprecipitation. The supernatants were incubated with a specific primary antibody, including IgG (Millipore Sigma, 17-620), anti-Flag (Millipore Sigma, F3165, RRID: AB_259529), and anti-Myc (Abcam, ab32, RRID: AB_303599) at 4 °C overnight with gentle rotation. The resulting mixture was incubated with protein A magnetic beads at 4 °C for 2 hours with gentle rotation. After the incubation, beads were precipitated, washed with IP lysis buffer, and eluted in IgG elution buffer (pH = 2, ThermoFisher) for 15 minutes at room temperature. For re-immunoprecipitation, the beads were eluted in a buffer containing 3×Flag peptides for 8 hours. The elution was used for the second round of immunoprecipitation. The final eluted liquids were collected for SDS-PAGE and immunoblotting.

SDS-PAGE and immunoblotting

After being washed with PBS, cells were lysed in ice-cold RIPA buffer containing 50 mM Tris (pH 7.5), 0.5% SDS, 150 mM NaCl, 0.5% NP40, 20 mM EDTA, 1 mM PMSF, and Protease Inhibitor Cocktail (1:200, Millipore Sigma, P8340) and sonicated on ice for 15 minutes. For human spinal cords, tissues were homogenized in a modified RIPA buffer (50 mM Tris [pH 7.5], 150 mM NaCl, 1% NP40, 0.1% SDS, 100 mM NaF, 17.5 mM β-glycerophosphate, 2.5% sodium deoxycholate, and 10% glycerol) containing phosphatase inhibitors 2 and 3 (1:100; Millipore Sigma), 1 mM PMSF, 2 mM $NaVO_4$, and Protease Inhibitor Cocktail (1:200, Millipore Sigma, P8340). After sonication on ice for 30 minutes, the samples were centrifuged at 12,000 g for 10 minutes at 4°C, and supernatants were collected. Protein concentrations were measured by the bicinchoninic acid assay (ThermoFisher, 23225), and protein levels of interest were detected by SDS-PAGE and immunoblotting. Membranes were incubated with primary antibodies in 5% BSA buffer at 4ºC overnight, including anti-UBQLN2 (Sigma, HPA006431, RRID:AB_1078707), anti-ILVBL (for human: Invitrogen, MA5-25585, RRID:AB_27232905; for mouse: Proteintech, 11220-1-AP, RRID:AB_2127187), anti-ALDH3A2 (Proteintech, 15090-1-AP, RRID:AB_2224316), anti-Flag (MilliporeSigma, F3165, RRID:AB_259529), anti-Ubiquitin (Cell Signaling Technology, 3933S, RRID:AB_2180538), anti-Myc (Abcam, ab32, RRID:AB_303599), anti-AMPK (Cell Signaling Technology, 5832, RRID:AB_10624867), anti-pAMPK(T172) (Cell Signaling Technology, 2535, RRID:AB_331250), anti-HA (Millipore Sigma, H6908, RRID:AB_260070), and anti-actin (Santa Cruz, sc-47778, RRID:AB_626632). After washes with TBST, membranes were incubated with a fluorescent secondary antibody at room temperature for 2 hours. Western blot images were captured by Odyssey scanner (LI-COR), and the OD intensity was analyzed with Image Studio (version 5.2.5) with normalization to actin.

Immunostaining and TUNEL staining

To prepare tissue slides for staining, mouse brains were isolated after perfusion with 4% paraformaldehyde, and cortical organoids were fixed in 4% paraformaldehyde. The fixed brains, lower spinal cords, and organoids were dehydrated in PBS containing 30% sucrose solution and cryosectioned using a freezing microtome (ThermoFisher Scientific, CryoSTAR NX70). Tissue slides were blocked and permeabilized in a buffer containing 5% normal serum and 0.3% Triton X-100 at room temperature for 2 hours. The fixed slides were incubated with primary antibodies: anti-Nestin (Santa Cruz, sc-23927, RRID: AB_627994), anti-PAX6 (BioLegend, 901302, RRID: AB_2565003), and anti-Tuj1 (GeneTex, GTX85469, RRID: AB_10629222), anti-VAMP2 (Proteintech, 10135-1-AP, RRID: AB_2256918), anti-NeuN (Cell Signaling Technology, 24307, RRID: AB_2651140), anti-GFP (Abcam, ab290, RRID: AB_2313768), cleaved caspase-3 (Cell Signaling Technology, 9664, RRID: AB_2070042) at 4ºC overnight. After being washed in PBS three times, slides were incubated with a fluorescent secondary antibody at room temperature for 2 hours, followed by PBS washes. Cell samples were washed twice with PBS and fixed with 4% formaldehyde for 20 minutes at room temperature. After being washed twice with PBS, the cells were permeabilized and blocked in a buffer containing 5% normal serum and 0.3% Triton X-100 in PBS for 2 hours at room temperature. Cells were incubated with primary antibodies: anti-ILVBL(Invitrogen, PA5-64335, RRID:AB_2642707), anti-ALDH3A2(Santa Cruz, sc-373921, RRID:AB_10918012), anti-Tom20 (Santa Cruz, sc-17764, RRID:AB_628381; Proteintech, 11802-1-AP, RRID:AB_2207530), anti-Flag (Sigma Aldrich, F1804, RRID: AB_262044), anti-ChAT (Millipore Sigma, AB143, RRID: AB_2079751), anti-Synaptophysin (Proteintech, 17785-1-AP, RRID: AB_365), anti-VAMP2 (Proteintech, 10135-1-AP, RRID: AB_2256918) at 4ºC overnight. The slides were washed and incubated with fluorescent secondary antibodies at room temperature for 2 hours, followed by PBS washes. For human tissue staining, slides were rinsed in xylene for 15 minutes twice and were sequentially dehydrated in 100% ethanol, 95% ethanol, 70% ethanol, 50% ethanol, and H_2O. Antigen retrieval was performed in buffer (0.1 M citrate acid and 0.1 M sodium citrate) at 95 °C for 30 minutes. After three washes with 1× PBS,

slides were incubated with blocking buffer (10% Normal donkey serum, 10% BSA, 0.3% Triton X-100) at room temperature for 1 hour, followed by incubation with antibodies of anti-TDP-43 antibody (Proteintech, 10782-2-AP, AB_615042), anti-UBQLN2 (Invitrogen, 35-4400, AB_2533204), and anti-PLIN2 (Proteintech, 15294-1-AP, AB_2878122) at 4 °C overnight. Slides were washed in 1× PBS and incubated with a secondary antibody for 1 hour at room temperature. After washing in 1× PBS three times, the slides were sealed with Prolong Gold Antifade reagent with DAPI (Invitrogen, P36931).

Apoptotic neurons in cortical organoids were measured by TUNEL staining (Elabscience, E-CK-A321) with co-immunostaining of Tuj1. Briefly, slides were fixed in 4% PFA buffer for 20 minutes at room temperature. After two washes in PBS, slides were incubated with Proteinase K at 37 °C for 10 minutes, followed by three washes of PBS. Slides with incubation of 100 μL DNase I (200 U/mL) or DNase I buffer were set as positive and negative controls, respectively. Slices were incubated with TdT equilibration buffer at 37 °C for 30 minutes and then 50 μL of Labeling working solution at 37 °C for 1 hour. After being washed in PBS three times, slides were subjected to immunostaining for Tuj1, as stated above. All slides, including cell and tissue slides, were sealed with a Prolong Gold Antifade reagent with DAPI (Invitrogen, P36931). Fluorescence images were captured with the SP8 confocal microscope (Leica). The fluorescence intensity per area or cell was analyzed by ImageJ.

Fatty acid oxidation and FA-driven mitochondrial respiration

FAO was measured using a commercial kit (BMR, E-141L/M/S), the principle of which is based on oxidation of fatty acyl-CoA, coupled to $FADH_2$/NADH-dependent reduction of INT to formazan, which exhibits an absorption maximum at 492 nm. Specifically, 5×10^6 HeLa cells or 1×10^7 iMNs were cultured under GS for 24 hours. After being washed with 1 × ice-cold PBS, cells were lysed in the ice-cold sample buffer (provided in the kit) for 5 minutes. The lysate was

centrifuged at ~14000 rpm for 5 minutes, and the supernatants were collected for measuring protein concentration using BCA. The protein concentrations of the samples were normalized to 2 mg/ml. Samples were mixed with FAO reaction buffer containing palmitoyl-CoA, octanoyl-CoA, or butyryl-CoA. After an incubation at 37 °C for 30-60 minutes, absorbance (O.D.) was measured at 492 nm using a plate reader. FAO activity in IU/L = ∆O.D. × 16.2 (30-minute incubation) or 8.1 (60-minute incubation).

Fatty acid oxidation (FAO)-dependent mitochondrial respiration was measured using the Mitochondrial Fatty Acid Oxidation Assay Kit (Abcam, ab222944) according to the manufacturer's protocol. On day 1, 1 × 10^4 HeLa cells were seeded in Seahorse XF assay plates and cultured in a complete medium. On the next day, cells were incubated in glucose-free medium overnight. Cells were washed twice with FA-free measurement medium and incubated in FAO assay medium supplemented with L-carnitine (5 μM), and mitochondrial respiration was monitored using a Seahorse XF Analyzer (Agilent), with sequential injections of oleate-BSA or 2-hydroxy-oleic acid (to stimulate FAO, 100 μM), oligomycin (to assess ATP-linked respiration, 2 μM), FCCP (to determine maximal respiration, 2.5 μM), and a rotenone (1 μM)/antimycin A (1 μM) mix (to measure non-mitochondrial respiration). OCR values were normalized to total protein. Potential OCR = maximum OCR − basal OCR. For specificity control, a parallel group was pretreated with etomoxir (10 μM), a CPT1 inhibitor that blocks mitochondrial fatty acid uptake. Basal, potential, and maximum mitochondrial respiration driven by fatty acids were statistically analyzed.

Supplementary Data Fig. 1

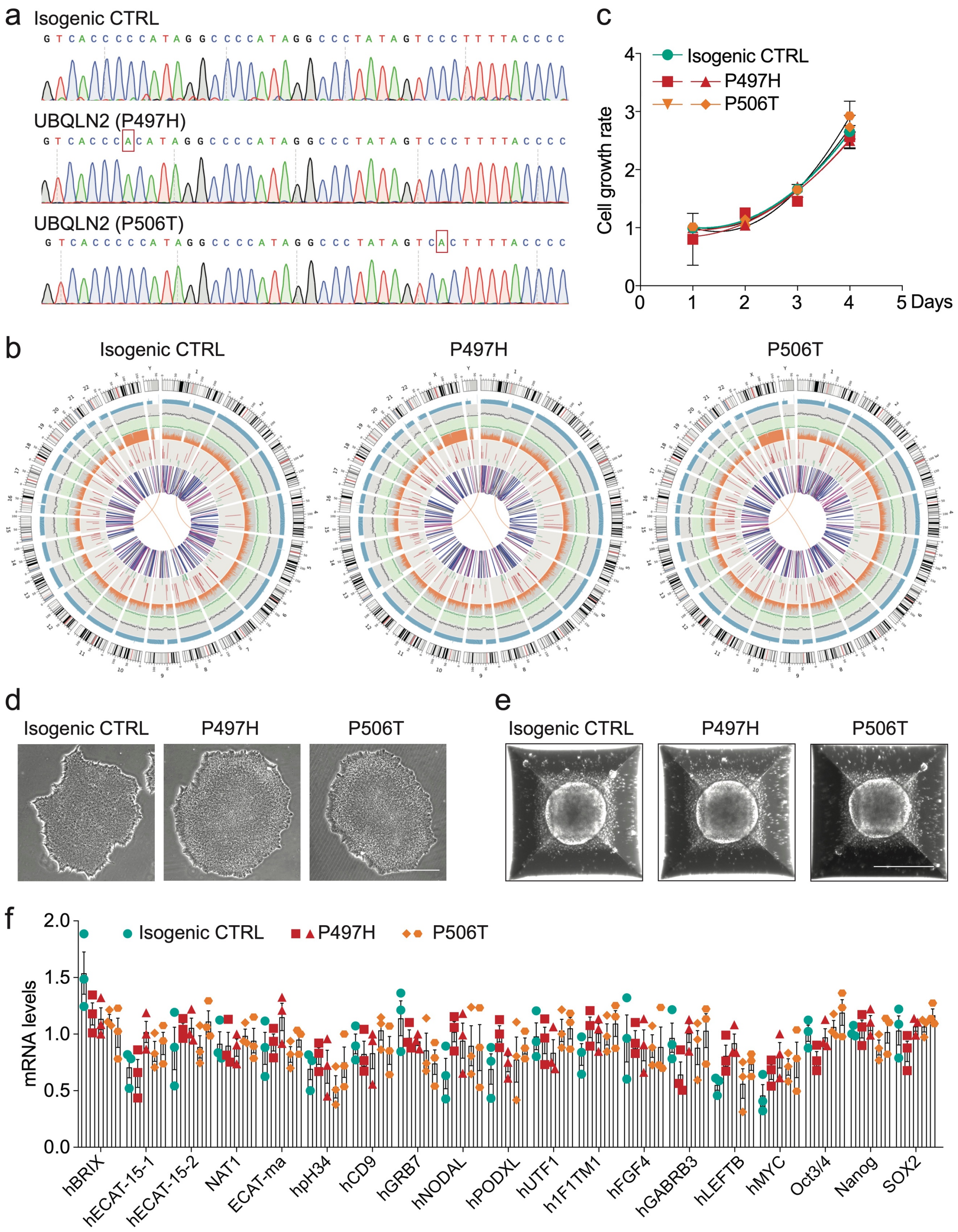

Supplementary Data Fig. 1: Characterization of engineered human iPSCs carrying disease-linked mutations in UBQLN2.

a, Sanger sequencing confirmed the successful CRISPR-mediated knock-in of P497H and P506T mutations in the UBQLN2 locus. **b**, Circos analysis of whole genome sequencing illustrating the genetic background of isogenic control and UBQLN2 mutant iPSCs. The outermost circle displays chromosome information. The second ring depicts read coverage as a histogram, with the average coverage calculated for each 0.5 Mbp region. The third ring shows InDel density as scattered black dots, each representing the number of InDels per 1 Mbp. The fourth ring indicates SNP density with green dots, each corresponding to the number of SNPs per 1 Mbp. The fifth ring presents the proportion of homozygous (orange) and heterozygous SNP (grey) in histogram style, calculated from each 1Mbp region. The sixth ring demonstrates CNV inference, with red indicating gains and green indicating losses. The innermost ring represents the structural variant inference in exonic and splicing regions, including BND (translocation, orange), INS (insertion, green), DEL (deletion, grey), DUP (tandem duplication, pink), and INV (inversion, blue). **c**, CCK-8 analysis of growth rate showed no significant changes among isogenic control and UBQLN2 mutant iPSCs (n = five biological replicates). **d** and **e**, Morphological observations and embryoid body formation of isogenic control and UBQLN2 mutant iPSCs showed no observable differences. **f**, Quantitative PCR analyses of stem cell markers indicated comparable expression levels among the groups (n = three biological replicates; one-way ANOVA followed by a Tukey's post-hoc test was applied for the statistical analyses with a threshold of adj_$p < 0.05$).

REFERENCES

1 Alexander, E. J. *et al.* Ubiquilin 2 modulates ALS/FTD-linked FUS-RNA complex dynamics and stress granule formation. *Proc Natl Acad Sci U S A* **115**, E11485-E11494, doi:10.1073/pnas.1811997115 (2018).

2 Park, I.-H., Lerou, P. H., Zhao, R., Huo, H. & Daley, G. Q. Generation of human-induced pluripotent stem cells. *Nature protocols* **3**, 1180-1186 (2008).

3 Takahashi, K. *et al.* Induction of pluripotent stem cells from adult human fibroblasts by defined factors. *cell* **131**, 861-872 (2007).

4 Felix Krueger, F. J., Phil Ewels, Ebrahim Afyounian, and Benjamin Schuster-Boeckler. FelixKrueger/TrimGalore: v0.6.7. Zenodo. (2021).

5 Kim, D., Langmead, B. & Salzberg, S. L. HISAT: a fast spliced aligner with low memory requirements. *Nature methods* **12**, 357-360 (2015).

6 Liao, Y., Smyth, G. K. & Shi, W. featureCounts: an efficient general purpose program for assigning sequence reads to genomic features. *Bioinformatics* **30**, 923-930, doi:10.1093/bioinformatics/btt656 (2014).

7 Love, M. I., Huber, W. & Anders, S. Moderated estimation of fold change and dispersion for RNA-seq data with DESeq2. *Genome Biol* **15**, 550, doi:10.1186/s13059-014-0550-8 (2014).
