# Supplementary material for: UBQLN2 links proteotoxicity with lipid metabolism in neurodegeneration
Source: Nat Neurosci. 2026 Mar 30;29(4):782–95. doi: 10.1038/s41593-026-02226-y (PMC13058728; doi:10.1038/s41593-026-02226-y)
Supplement: Supplementary file 2 — Reporting Summary [file 41593_2026_2226_MOESM2_ESM.pdf]

nature portfolio

Corresponding author(s): Jiou Wang

Last updated by author(s): Oct 30, 2025

Reporting Summary

Nature Portfolio wishes to improve the reproducibility of the work that we publish. This form provides structure for consistency and transparency in reporting. For further information on Nature Portfolio policies, see our Editorial Policies and the Editorial Policy Checklist.

Statistics

For all statistical analyses, confirm that the following items are present in the figure legend, table legend, main text, or Methods section.

n/a	Confirmed	
☐	☒	The exact sample size (*n*) for each experimental group/condition, given as a discrete number and unit of measurement
☐	☒	A statement on whether measurements were taken from distinct samples or whether the same sample was measured repeatedly
☐	☒	The statistical test(s) used AND whether they are one- or two-sided<br>*Only common tests should be described solely by name; describe more complex techniques in the Methods section.*
☐	☒	A description of all covariates tested
☐	☒	A description of any assumptions or corrections, such as tests of normality and adjustment for multiple comparisons
☐	☒	A full description of the statistical parameters including central tendency (e.g. means) or other basic estimates (e.g. regression coefficient) AND variation (e.g. standard deviation) or associated estimates of uncertainty (e.g. confidence intervals)
☒	☐	For null hypothesis testing, the test statistic (e.g. *F*, *t*, *r*) with confidence intervals, effect sizes, degrees of freedom and *P* value noted<br>*Give P values as exact values whenever suitable.*
☒	☐	For Bayesian analysis, information on the choice of priors and Markov chain Monte Carlo settings
☒	☐	For hierarchical and complex designs, identification of the appropriate level for tests and full reporting of outcomes
☒	☐	Estimates of effect sizes (e.g. Cohen's *d*, Pearson's *r*), indicating how they were calculated

Our web collection on statistics for biologists contains articles on many of the points above.

Software and code

Policy information about availability of computer code

Data collection	Dynamic SILAC DIA proteomics data was analyzed using Spectronaut (v.17.4) software. RNA-seq raw data were processed with TrimGalore v0.6.7 and controlled for quality with FastQC v.0.11.9. The processed reads were mapped to the human genome (hg38) and GENCODE v19 annotated transcripts using Hisat2 v2.2.1 and quantified with featureCounts v2.0.1. Raw gene counts were loaded into R to perform differential expression analysis with edgeR.
Data analysis	Most cellular experiments were performed in triplicate across two to three independent biological replicates. Statistical analyses were conducted using GraphPad Prism (v10.0) and R (v4.4.0). For multi-dimensional datasets (e.g., lipidomics, proteomics, and transcriptomics), differential analyses were performed using the limma package12, Spectronaut, or DESeq217, as appropriate. Multiple hypothesis testing corrections were applied using the Benjamini–Hochberg false discovery rate (FDR) procedure, with an adjusted p-value (adj_p) < 0.05 considered statistically significant.<br>For comparisons between two groups, the unpaired Mann-Whitney U tests or the paired two-tailed Student's t-tests were used. For analyses involving more than two groups, the Kruskal-Wallis test, ordinary one-way ANOVA, or Brown-Forsythe and Welch ANOVA test were applied with a Benjamini-Krieger-Yekutieli two-stage step-up procedure, Dunnett's multiple comparisons, or Dunn's post-hoc test. Two-way ANOVA followed by Tukey's multiple comparisons test was employed to assess the interaction effects of genotype and time or dose. Data distribution was assumed to be normal but this was not formally tested. All data were collected randomly and appropriately blocked. No statistical methods were used to predetermine sample sizes, but our sample sizes are similar to those reported in previous publications. Mice were randomly divided into groups receiving different treatments. No animals or data were excluded from the analyses.<br>Quantification of fluorescence images was performed using ImageJ. Fluorescence intensity or puncta counts were normalized to cell number, with each field represented as a single data point in graphs. Data are presented as mean ± SEM, or box plots are shown as minima to maxima.

For manuscripts utilizing custom algorithms or software that are central to the research but not yet described in published literature, software must be made available to editors and reviewers. We strongly encourage code deposition in a community repository (e.g. GitHub). See the Nature Portfolio guidelines for submitting code & software for further information.

Data

Policy information about availability of data

All manuscripts must include a data availability statement. This statement should provide the following information, where applicable:

- Accession codes, unique identifiers, or web links for publicly available datasets
- A description of any restrictions on data availability
- For clinical datasets or third party data, please ensure that the statement adheres to our policy

The omics data generated in this study are included in the supplementary materials (Supplementary Table 1-6). LC-MS/MS data are deposited in ProteomeXchange with a unique identifier, PXD048152. The raw RNA-seq data are deposited to the GEO database (GSE272994).

Research involving human participants, their data, or biological material

Policy information about studies with human participants or human data. See also policy information about sex, gender (identity/presentation), and sexual orientation and race, ethnicity and racism.

Reporting on sex and gender	All 19 human post-mortal spinal cord tissues were from TALS (TargetALS Postmortem Tissue Core) and VABBB (VA biorepository brain bank). Gender is not considered in this study. Informations regarding gender were provided in Supplementary Table 8.
Reporting on race, ethnicity, or other socially relevant groupings	All informations of humman samples regarding race, genotype, patient NO., clinical diagnosis, and age were provided in Supplementary Table 8.
Population characteristics	n/a
Recruitment	n/a
Ethics oversight	n/a

Note that full information on the approval of the study protocol must also be provided in the manuscript.

Field-specific reporting

Please select the one below that is the best fit for your research. If you are not sure, read the appropriate sections before making your selection.

☒ Life sciences ☐ Behavioural & social sciences ☐ Ecological, evolutionary & environmental sciences

For a reference copy of the document with all sections, see nature.com/documents/nr-reporting-summary-flat.pdf

Life sciences study design

All studies must disclose on these points even when the disclosure is negative.

Sample size	Sample size for each experiment was noted in the Figure Legends. No statistical method was used to predetermine the sample size. No statistical methods were used to predetermine sample sizes, but our sample sizes are similar to those reported in previous publications.
Data exclusions	No data were excluded from the statistical analysis.
Replication	All experiments were replicated successfully two-three times.
Randomization	The samples were allocated into experimental groups based on their genotypes or different treatments as described in Figure Legends.
Blinding	In the experiments involving animals, individual animals were coded and the investigators were blinded to the subject types during data collection. All the other molecular and cellular experiments were carried out following standard practice to ensure equal treatments of all samples.

Reporting for specific materials, systems and methods

We require information from authors about some types of materials, experimental systems and methods used in many studies. Here, indicate whether each material, system or method listed is relevant to your study. If you are not sure if a list item applies to your research, read the appropriate section before selecting a response.

Materials & experimental systems

n/a	Involved in the study
☐	☒ Antibodies
☐	☒ Eukaryotic cell lines
☒	☐ Palaeontology and archaeology
☐	☒ Animals and other organisms
☒	☐ Clinical data
☒	☐ Dual use research of concern
☒	☐ Plants

Methods

n/a	Involved in the study
☒	☐ ChIP-seq
☒	☐ Flow cytometry
☒	☐ MRI-based neuroimaging

Antibodies

Antibodies used	anti-UBQLN2 (Sigma, HPA006431, RRID:AB_1078707), anti-ILVBL (Invitrogen, MA5-25585, RRID:AB_27232905; Proteintech, 11220-1-AP, RRID:AB_2127187), anti-ALDH3A2 (Proteintech, 15090-1-AP, RRID:AB_2224316), anti-Flag (MilliporeSigma, F3165, RRID:AB_259529), anti-Ubiquitin (Cell Signaling Technology, 3933S, RRID:AB_2180538), anti-HA (Millipore Sigma, H6908, RRID:AB_260070), anti-Myc (Abcam, ab32, RRID:AB_303599), anti-AMPK (Cell Signaling Technology, 5832, RRID:AB_10624867), anti-pAMPK(T172) (Cell Signaling Technology, 2535, RRID:AB_331250), anti-actin (Santa Cruz, sc-47778, RRID:AB_626632), anti-Nestin (Santa Cruz, sc-23927, RRID: AB_627994), anti-PAX6 (BioLegend, 901302, RRID: AB_2565003), anti-Tuj1 (GeneTex, GTX85469, RRID: AB_10629222), anti-NeuN (Cell Signaling Technology, 24307, RRID: AB_2651140), anti-GFP (Abcam, ab290, RRID: AB_2313768), cleaved caspase-3 (Cell Signaling Technology, 9664, RRID: AB_2070042), anti-Rab5 (Cell signaling technology, 3547S, RRID: AB_2300649), anti-Flag (Sigma Aldrich, F1804, RRID: AB_262044), anti-ChAT (Millipore Sigma, AB143, RRID: AB_2079751), anti-Synaptophysin (Proteintech, 17785-1-AP, RRID: AB_2271365), anti-VAMP2 (Proteintech, 10135-1-AP, RRID: AB_2256918), anti-TDP-43 antibody (Proteintech, 10782-2-AP, AB_615042), anti-UBQLN2 (Invitrogen, 35-4400, AB_2533204), and anti-PLIN2 (Proteintech, 15294-1-AP, AB_2878122).
Validation	The validation or citations of the all primary antibodies for humans and/or mice have been provided on the manufacturers' websites.

Eukaryotic cell lines

Policy information about cell lines and Sex and Gender in Research

Cell line source(s)	HeLa cells and HEK293 cells were from ATCC. The human iPSCs carrying homozygous mutations of P497H or P506T were generated in collaboration with Synthego. The human iPSCs with or without TDP43 mutations were purchased from NINDS Human Cell and Data Repository and Jackson Lab. (cell line information was provided in Supplementary Table 7).
Authentication	For CRISPR Cas9-edited iPSCs, whole genome sequence were applied for measuring off-targets. The stemness of stem cell were measured by stem cell markers, embryonic formation. and neuronal differentiations. Mutations in human iPSCs were validated by Sanger sequencing. Motor neurons differentiated from human iPSCs were characterized by immunostaining against ChAT. Cortical organoids were characterized by cell markers for neuronal progenitor cells and matured neurons.
Mycoplasma contamination	All cell lines were regularly checked for mycoplasma contamination using a PCR-based detection kit (Millipore Sigma, MP0025).
Commonly misidentified lines (See ICLAC register)	No misidentified cell lines were used.

Animals and other research organisms

Policy information about studies involving animals; ARRIVE guidelines recommended for reporting animal research, and Sex and Gender in Research

Laboratory animals	C57BL/6 mice mice expressing wild-type and P506T human UBQLN2 driven by a Thy1.2 at the age of 52 weeks were used in this study. C57BL/6 mice received AAV-PhP.eB virus via retro-orbital sinus injection at the age of 8 weeks.
Wild animals	C57BL/6 mice
Reporting on sex	Both male and female mice were included, and gender was not considered in this study.
Field-collected samples	The study did not involve samples collected from the filed.
Ethics oversight	All animal procedures were performed at University of Maryland and Johns Hopkins University. All procedures were approved by the University of Maryland Baltimore Animal Care and Use Committees and conducted in full accordance with the NIH Guide for the Care and Use of Laboratory Animals.

Note that full information on the approval of the study protocol must also be provided in the manuscript.

Plants

Seed stocks	*Report on the source of all seed stocks or other plant material used. If applicable, state the seed stock centre and catalogue number. If plant specimens were collected from the field, describe the collection location, date and sampling procedures.*
Novel plant genotypes	*Describe the methods by which all novel plant genotypes were produced. This includes those generated by transgenic approaches, gene editing, chemical/radiation-based mutagenesis and hybridization. For transgenic lines, describe the transformation method, the number of independent lines analyzed and the generation upon which experiments were performed. For gene-edited lines, describe the editor used, the endogenous sequence targeted for editing, the targeting guide RNA sequence (if applicable) and how the editor was applied.*
Authentication	*Describe any authentication procedures for each seed stock used or novel genotype generated. Describe any experiments used to assess the effect of a mutation and, where applicable, how potential secondary effects (e.g. second site T-DNA insertions, mosiacism, off-target gene editing) were examined.*
