# Supplementary material for: UBQLN2 links proteotoxicity with lipid metabolism in neurodegeneration
Source: Nat Neurosci. 2026 Mar 30;29(4):782–95. doi: 10.1038/s41593-026-02226-y (PMC13058728; doi:10.1038/s41593-026-02226-y)

Fig. 4f

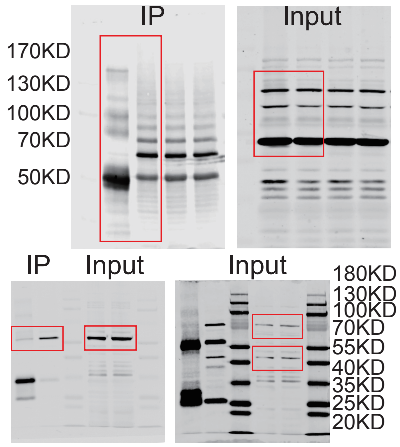

Fig. 4g

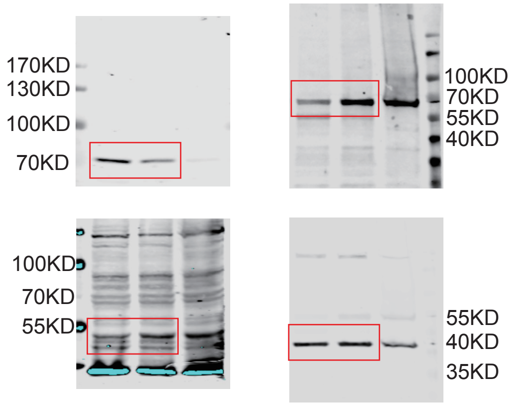

Fig. 6a

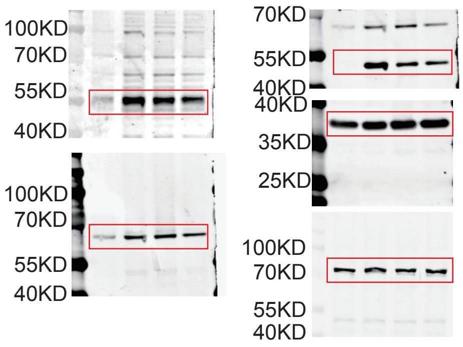

Fig. 6g

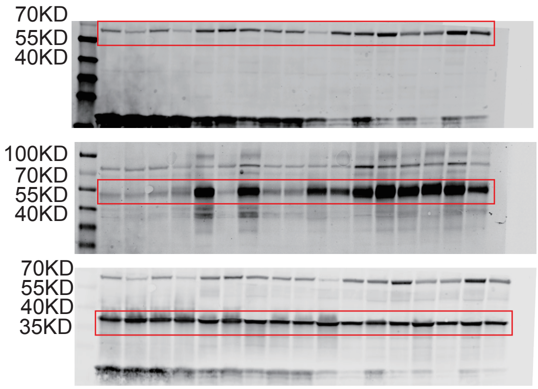

Fig. 6c

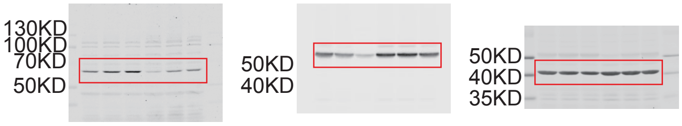

Extended Data Fig. 4c

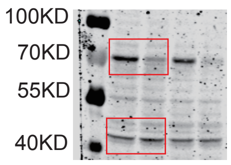

Extended Data Fig. 5h

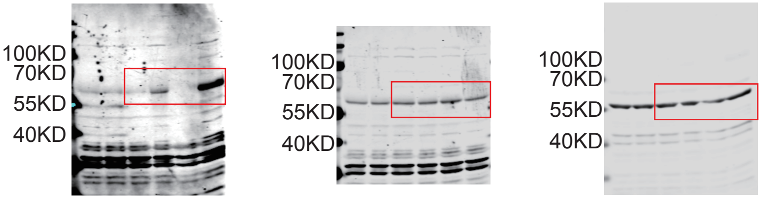

Extended Data Fig. 6a

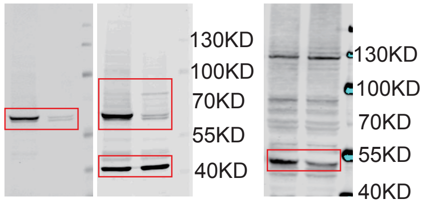

Extended Data Fig. 6b

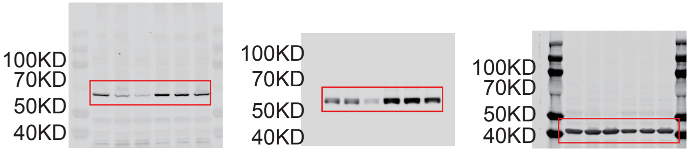

Extended Data Fig. 6c

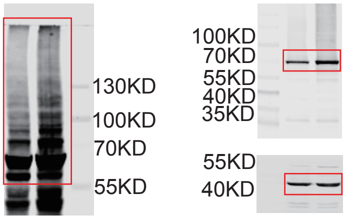

Extended Data Fig. 6f

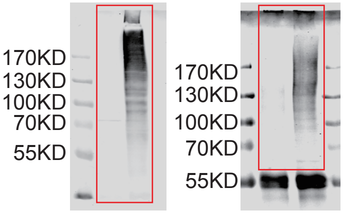

Extended Data Fig. 6j

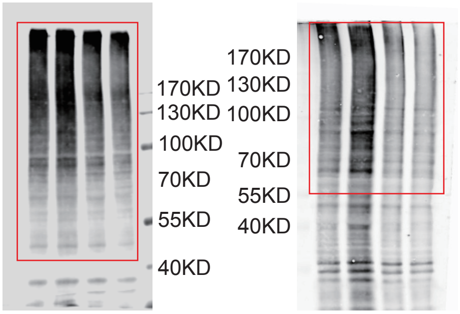

Extended Data Fig. 6g

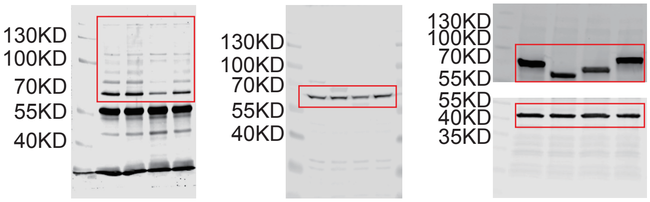

Extended Data Fig. 7a

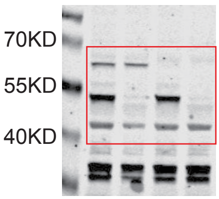

Extended Data Fig. 7b

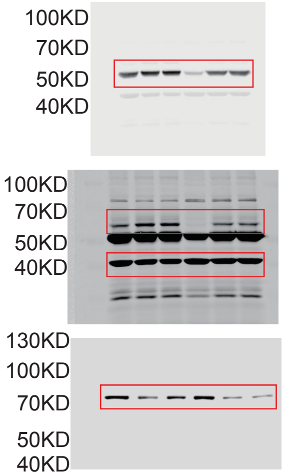

Extended Data Fig. 7d

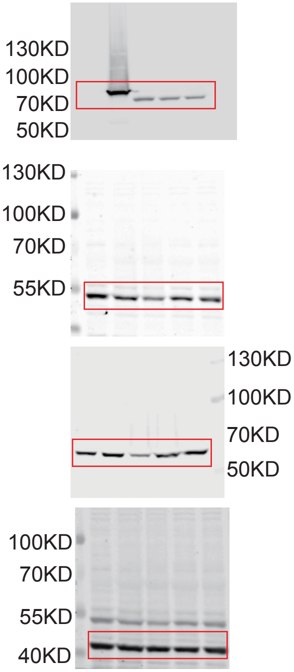

Extended Data Fig. 7e

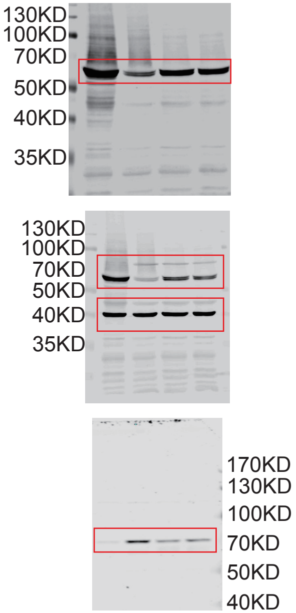

Extended Data Fig. 7f

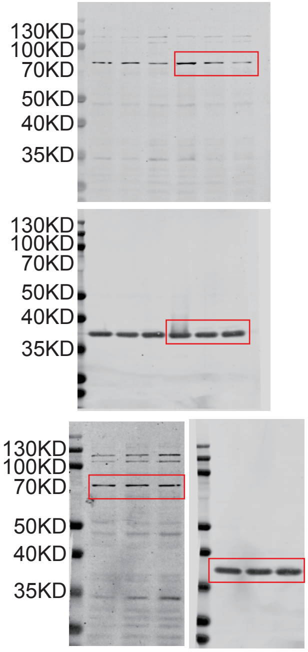

Extended Data Fig. 8i

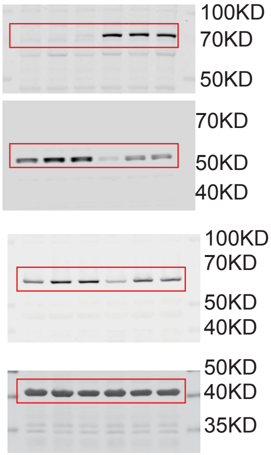

Extended Data Fig. 10e

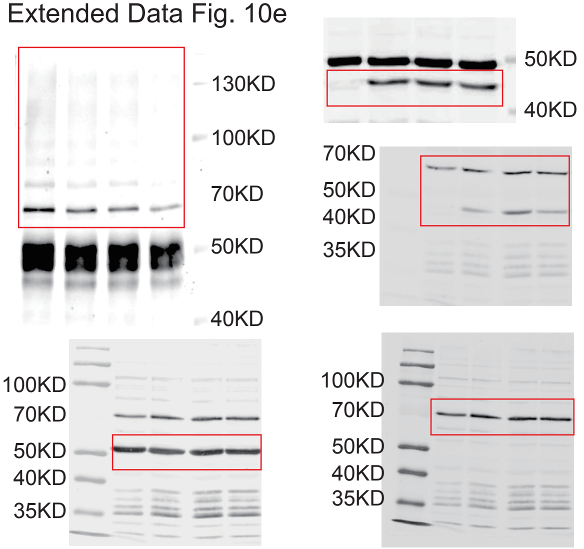

Extended Data Fig. 9e

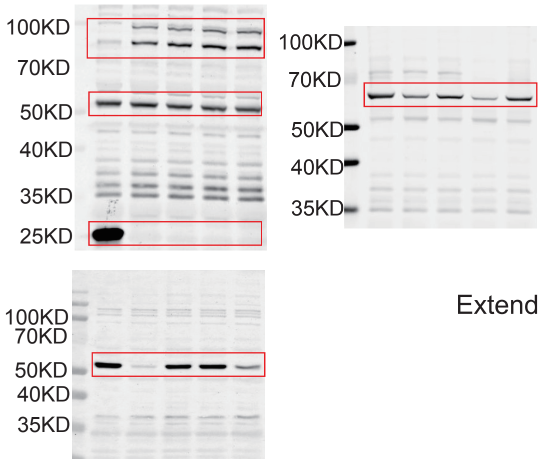

Extended Data Fig. 10f

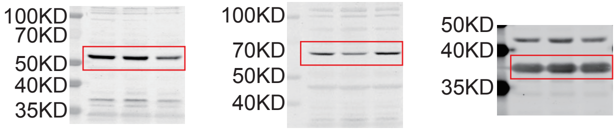

Supplement: Supplementary file 13 — Unprocessed western blots for Figs. 4 and 6 and Extended Data Figs. 4–10. [file 41593_2026_2226_MOESM13_ESM.pdf]
